# Trends in extraction and purification methods of Lignans in plant-derived foods

**DOI:** 10.1016/j.fochx.2025.102249

**Published:** 2025-02-03

**Authors:** Yu-tong Yang, Yuan Zhang, Yu Bian, Juan Zhu, Xue-song Feng

**Affiliations:** a School of Pharmacy, China Medical University, Shenyang, 110122, China; b Institute of Materia Medica, Chinese Academy of Medical Sciences and Peking Union Medical College, Beijing, 100050, China

**Keywords:** Deep eutectic solvent, Dispersive liquid-liquid microextraction, Dispersive micro solid phase extraction, Health foods, Hollow-fiber liquid-phase microextraction, Supramolecular solvent

## Abstract

Lignans are widely used as dietary supplements within health foods. However, excess addition of lignans can induce adverse reactions, therefore, it is necessary to develop rapid, effective, economical, and environmentally friendly extraction and purification methods to enhance lignan extraction efficiency. Recently, the advancement of sample pretreatment has been primarily directed towards the application of novel extraction solvents (e.g., supramolecular solvents) in dispersive liquid-liquid microextraction, the miniaturization of solid-phase extraction, the utilization of innovative adsorbent materials in dispersive solid-phase microextraction and matrix-assisted solid-phase extraction, and the employment of subcritical water extraction technology. Up to now, no systematic review has encompassed these advancements. Consequently, this review provides a comprehensive overview of the extraction and purification methods of lignans from plant-derived foods since 2017, with a particular focus on the application of microextraction technologies and new materials. It also analyzes the advantages and disadvantages of these methods and discusses their future developing trends.

## Highlights

• Different microextraction methods were summarized and compared.
• Supramolecular and deep eutectic solvent, as green solvents, were introduced.
• Different novel materials, such as molecularly imprinted polymers, were summarized.
• Magnetic nanomaterials as new adsorbent materials were introduced.
• The future trends in lignan pretreatment technologies are discussed.

## Graphical abstract

**Figure:**
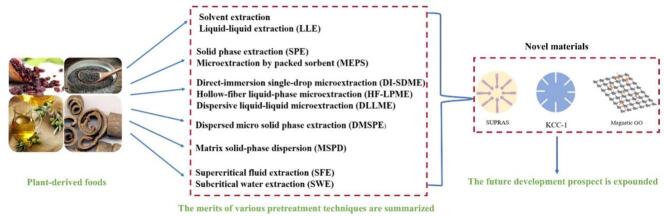

## 1 Introduction

Lignans, a class of polyphenols, are regarded as a notable group of phytoestrogens (Rodríguez-García, Sánchez-Quesada, Toledo, Delgado-Rodríguez, & Gaforio, 2019). In their natural state, lignans are primarily present in unbound forms, with some existing in conjunction with saccharides as glycosides (Wang et al., 2022). They are abundant in sesame and flaxseed, and can also be found in plant-derived foods such as wheat, oats, barley, and rye, as well as in soybeans, cruciferous vegetables like broccoli, and certain fruits like strawberries (Landete, 2012; Nadeem, Taj Khan, Khan, Shah, & Niaz, 2020; Rodríguez-García et al., 2019).

Lignans are typically synthesized via the shikimic acid pathway through the oxidative polymerization of phenylpropanoids, with four primary types depicted in Fig. S1 (Talapatra & Talapatra, 2015). The majority of lignans are composed of two-molecule phenylpropane backbones (C6-C3 monomer) that are formed via polymerization (Imai, Nomura, & Fukushima, 2006). Lignans are compounds that are linked by two cinnamic acids or cinnamyl alcohol through C-8-C-8′ bond, whereas neolignans do not share this connectivity (Moss, 2000). Lignans exhibit six distinct secondary structures based on their modes of junction and skeleton constructions (Fig.S2). The diverse functions of lignans depend on the modification of substituents on carbon atoms within the aromatic ring or the fatty hydrocarbon chain. Common substituents include hydroxyl, methoxy, and carboxyl groups (Yashin, Yashunskii, Vedenin, & Nifant'ev, N. E., Nemzer, B. V., & Yashin, Y. I., 2018). Table S1 provides essential data regarding selected representative lignans.

Due to their significant pharmacological properties, such as anti-inflammatory, antioxidant, anti-tumor, anti-fibrosis, neuroprotective, and metabolic regulatory effects (Zhou, Men, Sun, Wei, & Fan, 2021), lignans are frequently incorporated into health products. However, an excess addition of lignans may lead to adverse reactions, such as endocrine disorder and breast cancer (Socas-Rodríguez, Herrera-Herrera, Hernández-Borges, & Rodríguez-Delgado, 2017). Therefore, there is an urgent necessity to develop convenient, rapid, and reliable technologies to fully extract and enrich lignans in samples for accurate quantitative analysis. Moreover, many lignans in plant-derived foods remain unidentified. For instance, in the case of the extract of *Schisandra henryi* (*S. henryi*) added to health foods, researchers have primarily focused on extracting lignans from shoots and leaves, potentially neglecting other parts of *S. henryi* that might contain novel lignans (Jafernik, Ekiert, & Szopa, 2023). These unidentified novel lignans may exhibit significant biological activity, thus making the development of appropriate pretreatment methods indispensable.

Sample pretreatment allows for the extraction and purification of lignans from various complex matrices, which is essential for subsequent quantitative analysis. The appropriate pretreatment techniques can expedite experimental procedures, improve recovery efficiency, and decrease solvent consumption. Currently, the common pretreatment methods for lignans include solvent extraction and liquid-liquid extraction (LLE). In recent years, ultrasound-assisted extraction (UAE), pressurized liquid extraction (PLE), microwave-assisted extraction (MAE), and microextraction techniques have been developing rapidly.

Since 2017, only one publication has been centered around the advancements in lignan pretreatment. (Patyra, Kołtun-Jasion, Jakubiak, & Kiss, 2022) overviewed both common and alternative methods for the extraction and isolation of lignans from plant materials, including Soxhlet, maceration, digestion, UAE, PLE, MAE, and supercritical fluid extraction (SFE). They demonstrated that these alternative methods, like UAE, PLE, MAE, and SFE, have expedited extraction speed, enhanced extraction efficiency, diminished solvent consumption, and contributed to environmental conservation. Building upon this, a range of innovative pretreatment methods have been developed and implemented, with a specific emphasis on the progress in microextraction techniques and the application of novel materials. When applied to lignans within diverse matrices, these methods enhance convenience, speed, environmental sustainability, and efficiency. As a result, this paper presents a comprehensive overview of pretreatment methods for lignans in plant-derived foods, meticulously analyzing approximately seventy published studies (The search criteria are detailed in Supplementary Material). In this study, different pretreatment methods, such as liquid phase microextraction (LPME), dispersive micro solid phase extraction (D-μ-SPE), supercritical fluid extraction (SFE), and matrix solid-phase dispersion (MSPD), have been summarized and compared. In addition, the advantages and disadvantages of these techniques have been deliberated, and future perspectives have been explored. Notably, our overview places particular emphasis on the novel materials and solvents employed in pretreatment methods.

## 2 Extraction and purification methods for lignans

The existence of impurities within the matrix, including proteins, lipids, saccharides, and mineral salts, can reduce the sensitivity of determinations, impair the accuracy of analysis, and even cause damage to equipment. Therefore, it is crucial to conduct appropriate extraction and purification procedures before analysis.

### 2.1 Commonly used pretreatment methods for lignans

Frequently employed techniques for extraction and purification of lignans encompass solvent extraction, dilution, centrifugation, filtration, hydrolysis, and protein sedimentation. These methods offer the advantages of ease of use and safe handling without the need for expensive equipment. Recently, in order to reduce solvent usage and processing time, several updated methods have been developed, including solvent extraction with assisted techniques (e.g., UAE, MAE, and enzyme-assisted extraction (EAE)), solvent extraction using novel solvents (e.g., ionic liquids (ILs), hybrid ionic liquids (HILs), and deep eutectic solvent (DES)), PLE, and LLE.

UAE utilizes cavitation, quake, pulverization, and stirring to facilitate the penetration of the solvent into natural cells for lignan extraction (Wen et al., 2018). MAE triggers violent oscillations of biological water molecules, breaking the intermolecular hydrogen bonds in cell membranes and accelerating the release of lignans from cells or solutions into the solvent (Zhang, Cai, Cheng, & Zhang, 2022). UAE and MAE can immensely enhance extraction efficiency by facilitating the contact areas between lignans and solvents. EAE is targeted at hydrolyzing the cell wall, promoting the rapid release of lignans through the high efficiency, specificity, and regioselectivity of enzymes, without destroying other substances except the substrates, thereby reducing sample impurities and batch-to-batch discrepancies. EAE has demonstrated high extraction efficiency (Nadar, Rao, & Rathod, 2018). Angeloni et al. (2020) developed an EAE method for the extraction of secoisolariciresinol, matairesinol, lariciresinoland, and other nine compounds in green coffee. In this study, clara-amylase and taka-amylase were compared and the former was selected since it gave a better extraction efficiency. Under optimal conditions, satisfactory recoveries (87–97 %) were obtained. In addition, EAE methods are often praised for superior selectivity and being environmentally friendly.

For lignans in solid or semi-solid samples, solvent extraction is usually the initial step. Commonly used extraction solvents include methanol (Chen et al., 2018; Zhou et al., 2021), ethanol (Olmo-Garcia et al., 2018; Wang et al., 2020), and acetone (Ma et al., 2019; Nikule et al., 2020). Nowadays, many novel extraction solvents have also been contrived, including ILs, HLs, and DES. ILs are attracting significant attention as green solvents. Composed of organic cations and inorganic or organic anions, ILs possess special properties such as high thermal stability, tunable chemical structure, and outstanding solubilization properties for both organic and inorganic lignans (Ferraz, Branco, Prudêncio, Noronha, & Petrovski, 2011; Xia et al., 2020; Yang et al., 2018). A prominent advantage of solvent extraction based on ILs is their high extraction efficiency. Guan, Luo, Liang, and Yu (2018) developed a solvent extraction based on ILs to isolate schisandrin A, schisantherin A, and deoxyschizandrin from health foods. In this study, [C_6_MIM][BF_4_], an IL, was chosen as the extraction solvent. After optimization, satisfactory recoveries between 74.19 % and 109.33 % were gained. Moreover, solvent extraction based on ILs has always been praised for being environmentally friendly. In the study mentioned above, only a minimal amount of 200 μL ILs was required (Guan et al., 2018). To improve the properties of common ILs, Lewis alkaline anions have been adopted to update common ILs. ILs containing Lewis base, with alkaline sites, can exhibit catalytic activity, hydrolyzing bound biphenyl cyclooctene lignans to a free state, thus accelerating the extraction process (Ma et al., 2011; MacFarlane, Pringle, Johansson, Forsyth, & Forsyth, 2006). The major merit of solvent extraction based on ILs containing Lewis base is its time-saving nature. Xia et al. (2020) established solvent extraction based on ILs containing Lewis base to extract schizandri, schisantherin A, deoxyschizandrin, γ-schizandrin, and schisandrin C in *Schisandra chinensis* (*S.chinensis*) fruits. In this study, [C4mim]Ac (containing Lewis base) was selected as extraction solvent. Under optimal conditions, the total extraction time was 12 min. In addition, solvent extraction based on ILs containing Lewis base also has the merits of high recoveries and being environmentally friendly. However, some plant-derived foods contain various lignans with considerable polar diversities, which can hinder concurrent extraction (Wu, Wu, Wu, Wang, & Tan, 2021). Thus, HILs have been developed, using ILs with a high lignan extraction rate and mixing them in an optimal ratio to extract a broader range of lignans (Wu et al., 2021). Solvent extraction based on HILs has shown advantages in terms of high extraction efficiency. Wu et al. (2021) employed solvent extraction based on HILs to extract eighteen multi-polar lignans in health foods. In this experiment, HILs ([AMIM]Cl:[EMIM][BF4]:[EMIM][OAc] = 1:5:5) and ethanol were compared, and the former was chosen since it gave a better recovery. After optimization, the content of total lignans was 69 mg/g, 47 % higher than that of the ethanol system. DES is also a green solvent composed of a hydrogen bond acceptor (e.g., quaternary ammonium salt) and a hydrogen bond donor (e.g., acylamino, carboxylic acid, polyhydric alcohol) at a specific ratio (Xue, Wang, Chen, Hu, & Bai, 2019). Currently, DES can be classified into five types: Type I (quaternary ammonium salts-metal chlorides), Type II (quaternary ammonium salts-metal hydrates), Type III (quaternary ammonium salts-various organic compounds), Type IV (metal halides‑hydrogen bond donor), and Type V (comprised of non-ionic substances, such as aldehyde-amine) (Abranches et al., 2019; Bişgin, 2024; Gao et al., 2024; Kaçanbüre & Bişgin, 2025). The composition ratio of DES, the volume of DES, pH, and vortex time can significantly affect the extraction efficiency of lignans (Bişgin, 2023). Solvent extraction based on DES exhibits extremely high extraction efficiency. Chen et al. (2024) employed solvent extraction based on DES to extract Schizandrol A, schizandrol B, schisantherin A, schisandrin A, and schisandrin B from health foods. In this study, the ratio of choline chloride to glycolic acid was 4:1, and the solid–liquid ratio was 1:20 (W_g_: V_mL_). Under optimal conditions, the recovery rates of the five lignans were 4.019–10.89 mg/g, which is 2.57 times higher than those of solvent extraction based on ILs. In addition, solvent extraction based on DES has also shown the advantages of reducing experimental time. In the study mentioned above, the total experimental time was merely 20 min (Chen et al., 2024).

PLE, also called accelerated solvent extraction (ASE), is an automated technique for extracting solid or semi-solid samples using organic solvents at elevated temperatures (50–200 °C) and pressures (1000–3000 PSI) (Mustafa & Turner, 2011). PLE is often praised for its high extraction efficiency. Cea Paze et al. (2019) established a PLE method for extracting pinoresinol and acetoxypinoresinol from olive oil by-product. In their work, the temperature and pressure were set to 108 °C and 1500 psi, respectively. After optimization, the contents of pinoresinol and acetoxypinoresinol were 10.9 ± 0.3 and 37.8 ± 0.8 mg/g dry weight, respectively. In addition, PLE has also shown advantages in terms of time-saving. In the study mentioned above, the whole extraction took only 20 min, significantly shorter than that of the commonly used solvent extraction methods (Cea Paze et al., 2019).

LLE is another commonly used method for extracting lignans from different samples (Zhang, Dai, Xiao, & Liu, 2023). When two completely or partially immiscible phases come into contact, LLE transfers lignans from the sample solution to the solvent. Commonly used LLE extractants for lignans in aqueous samples include ethyl acetate (Gao et al., 2018; Mattioli et al., 2017), ether (Liu et al., 2017; Mattioli et al., 2019), chlorinated alkanes (Fakhari et al., 2019; Sykłowska-Baranek et al., 2018), acetone (Mangindaan et al., 2017; Wu et al., 2020), and methyl tert-butyl ether (Bess et al., 2020; Kim et al., 2017), while extractants for lignans in oil samples include methanol or ethanol aqueous solution (Caprioli, Boarelli, Ricciutelli, Sagratini, & Fiorini, 2019) and petroleum ether (Yashaswini, Sadashivaiah, Ramaprasad, & Singh, 2017). LLE has shown merit in terms of high extraction efficiency. Gao et al. (2018) used LLE method to extract eight lignans from *Arctium lappa* L. fruit. In this work, ethyl acetate was selected as the extractant. Under optimal conditions, the total lignan content, with arctigenin as a reference, was 84.7 %. Moreover, the merits of LLE include its simplicity, ease of operation, and low cost.

In commonly used pretreatment methods for lignans, solvent extraction continues to be a widely used method for extracting lignans. Many green solvents (e.g., ILs, HILs, and DES) and assisted techniques (e.g., UAE, MAE, EAE, and PLE) are applied in solvent extraction to minimize solvent usage and enhance extraction efficiency, thereby making the solvent extraction more environmentally friendly. LLE is also acknowledged for its simplicity in extracting and purifying of lignans, although it requires a significant volume of solvent.

### 2.2 Liquid-phase microextraction (LPME)

LPME, also called liquid-liquid microextraction (LLME), is one of the novel sample pretreatment technologies. Derived from LLE, LPME is a miniaturized version that mitigates the high solvent consumption and enhances the enrichment of trace lignans, thus addressing the limitations of LLE (Han & Row, 2012). LPME enhances the recovery and enrichment by using less solvent (a few microliters-dozens of microliters) and less time, in line with the principles of green chemistry (Hu, Chen, & Yang, 2019). In recent years, various LPME methods, including direct-immersion single-drop microextraction (DI-SDME), hollow-fiber liquid-phase microextraction (HF-LPME), and dispersive liquid-liquid microextraction (DLLME), have been developed and employed for the extraction of lignans (See Table 1). (An, He, Guo, & Dong, 2021; Geng, Chen, Li, Bai, & Hu, 2020; Li et al., 2018a; Li, Hu, Chen, & Bai, 2017; Li, Hu, Chen, Wang, & Bai, 2018; Liu, Zhang, Qin, & Yu, 2017; Liu, Zhang, Yang, & Yu, 2019; Qin et al., 2023; Wang et al., 2019; Xue et al., 2019; Xue, Yang, Chen, Bai, & Hu, 2021).

**Table 1:** LPME methods for lignans in food.

Sample	Target analytes	LPME methods	Extraction solvent	LPME process	LPME results	Reference
*Forsythia suspensa* fruit	Arctiin; Phillyrin; Arctigenin; Phillygenin	VA-DLLME	5 mmol/L Tetrabutylammonium bromide (TBAB)/*n*-hexanol (SUPRAS)	① Soak 0.5 g of sample powder in 70 % methanol (25 mL) for 12 h ② Ultrasound for 30 min ③ Adjust the pH to 4, add 700 μL of SUPRAS ④ Vortex for 30 s ⑤ Centrifuge at 4000 rpm for 3 min ⑥ Transfer the supernatant for analysis.	Average recoveries: 96.5–104.8 % EF: 6–170	(Qin et al., 2023)
Health foods	Magnolol; Honokiol	BT-OIS-LPME	N-hexanol (immobilized with 15 % (*w*/*v*) of NaCl on the surface)	① Prepare BT by filling it with n-hexanol and immersing it in a 15 % ② NaCl solution for 10 min ③ Soak 2.0 g of sample in 30 mL of methanol ④ Ultrasound for 45 min ⑤ Centrifuge with a magnetic at 1200 rpm for 30 min at room temperature ⑥ Rinse the BT cavity with 20 μL methanol.	Average recoveries: 84.5–99.8 % EFs: 73–76	(Wang et al., 2019)
Health foods	Magnolol; Honokiol	HFCF-UF	Methanol	① Dilute 1 mL of samples to 10 mL with methanol ② Insert the HFCF-UF device ③ Centrifugate for 15 min at 3.8 × 10^3^ g ④ Withdraw the solution from the hollow fiber for analysis	Average recoveries: 96.8–97.7 %	(An et al., 2021)
Health foods	Magnolol; Honokiol	Hollow fiber cell fishing	The cell suspension containing the human renal tubular ACHN cell line, hepatoma HepG-2 cell line and human cervical cancer cell line HeLa	① Inject 1 mL of cell suspension into the fiber ② Culture for 24 h, immerse the fiber in the sample ③ Extract at 37 °C and 600 rpm for 3 h using a magnetic stirrer ④ Elute with 40 μL of methanol ⑤ Centrifuge the eluant at 12000 rpm for 20 min ⑥ Transfer the supernatant for analysis.	MG and HK were effectively extracted	(Li, Hu, et al., 2018)
Health foods	Magnolol; Honokiol	OIS-HF-LPME	N-hexanol/n-heptanol = 2:8 v:v	① Clean HF ② Immerse HF in extraction solvent for 30 s ③ Immerse HF in 15 % NaCl w/v for 30 min ④ Bend HF into U-shaped ⑤ Immerse HF in biological samples ⑥ Extract at 1200 rpm for 45 min at room temperature using a magnetic stirrer ⑦ Elute with 40 μL of methanol ⑧ Centrifuge the eluant at 12000 rpm for 10 min ⑨ Transfer the supernatant for analysis.	Average recoveries: 103.2–109.5 % EF: 29.3–29.6	(Li et al., 2018)
Health foods	Magnolol; Honokiol	OIS-HF-LPME	N-hexanol/n-heptanol = 2:8, v:v	① Clean HF ② Immerse HF in the extraction solvent for 30 s ③ Immerse HF in NaCl of 15 % w/v for 30 min ④ Bend HF into U-shaped ⑤ Immerse HF in biological samples ⑥ Extract at 1200 rpm for 45 min at room temperature using a magnetic stirrer ⑦ Elute with 40 μL of methanol ⑧ Inject 20 μL solution into the analysis system	Average recoveries: 94.1–98.2 % EF: 43.1–67.3	(Li et al., 2017)
Health foods	Magnolol; Honokiol	SFOD-LPME	1-Undecanol/1-dodecanol = 3:7, v/v	① Mix samples with 20 mL of methanol, shake ② Ultrasound for 45 min, centrifuge at 2500 rpm for 4 min ③ Dilute the supernatant 50-fold with double-distilled water ④ Place 7 mL of supernatant and 20 μL of extraction solvent in a glass vial ⑤ Extract with a magnetic stirrer at 1200 rpm for 30 min Freeze at −20 °C for 6 min ⑥ Use a small spoon to remove the solidified aggregates for analysis.	Average recoveries: 97.5–100.0 % EF: 229–252	(Geng et al., 2020)
Health foods	Magnolol; Honokiol	VA-DLLME	TBAC/hexanoic acid = 1:2, v:v (DES6)	① Soak samples in 30 mL of methanol for 12 h ② Ultrasound for 45 min ③ Filter the solution ④ Dilute with 6.49 mL of pH 2 HCl ⑤ Add 300 μL of DES6 ⑥ Vortex for 2 min ⑦ Centrifuge for 2 min at 3000 rpm ⑧ Collect DES6 for analysis.	Average relative recoveries: 98.2–97.7 % EF: 197–210	(Xue et al., 2021)
Health foods	Magnolol; Honokiol	3p-HF-LPME	1. Extraction phase solvent: n-heptanol/n-nonanol =7:3, v:v 2. Acceptor phase solvent: MTAC-glycerol/methanol = 9:1, v:v	① Clean HF ② Immerse HF in the extraction solvent for 30 s ③ Blow out solvent ④ Inject the DES into the HF ⑤ Place 0.14 mL of samples and 6.86 mL of HCl (pH = 2) in vial ⑥ Bend HF into U-shape ⑦ Extract with a magnetic stirrer at 1200 rpm for 45 min ⑧ Flush HF with 20 μL of methanol ⑨ Vortex the mixture for 30 s ⑩ Transfer the supernatant for analysis	Average recoveries: 94.2–97.6 % EF: 71	(Xue et al., 2019)
Sesame oil	Sesamin; Sesamolin; Sesamol	UALLME	Choline chloride/p-cresol = 1:2, v:v (DESs)	① Mix 0.20 g of sesame oil with 3 mL of n-hexane ② Add 400 μL of DESs ③ Sonicate for 30 min at 50 °C ④ Centrifuge for 5 min at 4000 rpm ⑤ Transfer DES for analysis	Recoveries: 97.3–120 %	(Liu et al., 2019)
Sesame oil	Sesamol	UALLME	choline chloride/ethylene glycol = 1:2, mol/mol (DESs)	① Mix 0.20 g of sesame oil with 5 mL n-hexane ② Add 400 μL of DESs ③ Sonicate for 30 min ④ Centrifugate for 10 min at 3000 rpm ⑤ Transfer DES for analysis	Recoveries: 97–108 %	(Liu, Zhang, et al., 2017)

#### 2.2.1 DI-SDME methods for lignans

**Fig. 1:**
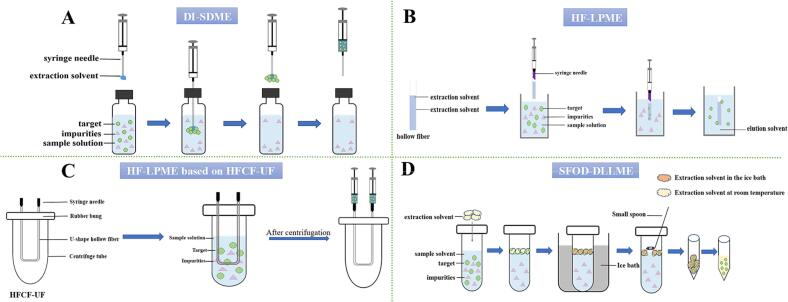
Flow chart of 4 novel liquid-phase microextraction technologies.

DI-SDME was first put forward by Jeannot and Cantwell (1996). The detailed procedures of the common DI-SDME are depicted in Fig. 1A. Commonly used DI-SDME solvents include n-hexanol, toluene, and hexane (Farajzadeh, Sorouraddin, & Mogaddam, 2014; Malczak & Gajda, 2023). In recent years, some researchers have devoted themselves to updating common solvents and have achieved remarkable results. Oil-in-salt (OIS) is an updated solvent, whose formation method involves adding salt into the organic solvent. The principle underlying this is that the salting-out effect enhances the solubility of weak electrolytes in the organic solvent while diminishing their solubility in the aqueous solvent (Wang et al., 2019). DI-SDME based on OIS has always been praised for its high enrichment ratio, making it advantageous for the extraction of trace lignan. Wang et al. (2019) employed DI-SDME based on OIS to extract trace amount of magnolol (MG) and honokiol (HK) from health foods. In this study, they immersed a vessel containing n-hexanol into 15 % (*w*/*v*) of NaCl solution for 10 min to form OIS. Under optimum conditions, satisfactory enrichment factors (EFs) (73 for HK and 76 for MG) were obtained. Moreover, DI-SDME based on OIS has shown an advantage in outstanding extraction efficiency (EE), in the study of Wang et al. mentioned above, the EEs were 23.4 % (HK) and 24.3 % (MG), respectively (Wang et al., 2019).

In addition to the extraction solvent, the supporting vessel for the droplet also plays an important role. Commonly used supporting vessels include micro syringe needle and capillary orifice (Jain & Verma, 2020; Yıldırım, Cocovi-Solberg, Uslu, Solich, & Horstkotte, 2022). However, the droplet is prone to falling off or breaking when using a common vessel, thereby compromising the extraction process (Ramos Payán, Bello López, Fernández-Torres, Callejón Mochón, & Gómez Ariza, 2010). Consequently, several techniques have been developed to protect the drop. Using a ballpoint tip (BT) as a protective container for placing organic extractant droplets into the cavity is a valid method (Ji et al., 2018). DI-SDME based on BT has been widely praised for enhancing extraction success rates and minimizing the contamination of droplets by matrices and impurities (Wang et al., 2019).

#### 2.2.2 Hollow-fiber liquid-phase microextraction (HF-LPME)

HF-LPME was first introduced in 1999 (Pedersen-Bjergaard & Rasmussen, 1999). The aperture of the hollow fiber, which protects the organic solvents, is narrow (about 0.2 μm), thus preventing the entry of macro-molecular impurities and facilitating the extraction of lignans from plant-derived foods (Ramos Payán et al., 2010). The operating procedure of HF-LPME is shown in Fig. 1B. HF-LPME has been consistently recognized for its excellent enrichment ratio. Xue et al. (2019) developed an HF-LPME for the extraction of MG and HK from health foods. In this study, methyltrioctylammonium chloride/glycerol (1,3, n/n) was selected as the solvent. Under optimal conditions, the LOD ranged from 0.3 to 0.5 ng/mL, and the LOQ was in the range of 1.0–1.4 ng/mL. In addition, HF-LPME also exhibits the advantage of shortening the experimental time and improving the extraction efficiency.

Extraction solvents are essential for achieving accurate selectivity and high enrichment efficiency (Hu et al., 2022). In recent years, plenty of new solvents have been developed to advance the technology of HF-LPME. Commonly used HF-LPME solvents include DES and OIS. DES possess numerous properties, such as biodegradability and excellent thermal stability (Xue et al., 2019). HF-LPME based on DES has always been commended for its high enrichment factor. Xue et al. (2019) developed an HF-LPME based on DESs (methyltrioctylammonium chloride/glycerol, 1:3, n/n) for extracting MG and HK from health foods. In this study, n-hexanol/n-heptanol (2:8, *v*/v) and DESs were compared as solvents, and the latter was selected due to its superior enrichment effect. Under optimal conditions, HF-LPME based on DESs obtained a higher EF (71), which was 37.5–53 higher than that of the common method. In addition, HF-LPME based on DESs has also demonstrated advantages in high extraction efficiency and selectivity, low budget, and environmental friendliness. OIS is also a commonly used solvent, now widely applied in HF-LPME. OIS can decrease the negative effects caused by ion enhancement due to the salting out effect, thus increasing the analytic signals of lignans. One merit of using an HF-LPME based on OIS is its ability to enrich and concentrate a diverse range of lignans. Li et al. (2018b) developed an HF-LPME based on OIS to extract HK and MG from health foods. In this study, a mixture of n-hexanol and n-heptanol (2,8, v/v) was selected as the oil phase, and 5 % NaCl (*w*/*v*) was used as the salt membrane. After optimization, the EFs were determined to be 29.3 for MG and 29.6 for HK. Furthermore, HF-LPME based on OIS demonstrated good extraction efficiency. In the mentioned above, the average recovery rates were 103.2 ± 8.0 % for MG and 109.5 ± 1.1 % for HK (Li, Hu, et al., 2018).

As is widely recognized, sample pretreatment steps have an impact on the precision of results: the more steps there are, the higher the potential for error. Therefore, these steps have been optimized (Jiang, Jaroch, & Pawliszyn, 2023). Researchers have developed a device that uses a centrifuge fiber and a U-shape hollow fiber, thereby reducing the extraction process for lignans to a single step. This device is known as hollow fiber centrifugal ultrafiltrate (HFCF-UF) (An et al., 2021). The detailed processes are shown in Fig. 1C. A significant advantage of using HF-LPME based on HFCF-UF is that it saves time. An et al. (2021) developed an HF-LPME method based on HFCF-UF for extracting MG and HK from health foods. In this study, they utilized a polypropylene hollow fiber. After optimization, the entire process took less than 15 min. Meanwhile, HF-LPME based on HFCF-UF has shown advantage in terms of excellent recoveries. In the above-mentioned study, high recoveries (92.6–101.7 %) for MG and HK were obtained (An et al., 2021).

#### 2.2.3 Dispersive liquid-liquid microextraction (DLLME)

DLLME was first proposed by Rezaee et al. (2006). DLLME is an easy and quick microextraction technology that utilizes an appropriate amount of microliters of extraction solvent and a milliliter of dispersant to extract lignans from plant-derived foods (Leong, Fuh, & Huang, 2014). Commonly used DLLME solvents include chlorobenzene, chloroform, and carbon disulfide; commonly used dispersants include methanol, acetonitrile, and acetone (Hu et al., 2019). One merit of DLLME is its high enrichment efficiency. Guo, Pang, Zhang, Jiang, and Pang (2013) developed DLLME to extract MG and HK from health foods. In this experiment, 1,2-dichloroethane was employed as the extraction solvent, and tetrahydrofuran as the dispersant. Under optimum conditions, superior EFs, 87 for MG and 119 for HK, were obtained. Likewise, DLLME has shown merits in terms of good extraction efficiency. In the study mentioned above, the recoveries were 90.2–96.7 % for MG and 94.4–99.4 % for HK (Guo et al., 2013).

In the process of DLLME, the extraction solvent is a key area for advancement, considering that common solvents are often toxic and the solvent significantly influences the extraction outcome, thus prompting the development of many novel solvents (Leong et al., 2014). In recent years, different novel DLLME solvents for lignans include supramolecular solvent (SUPRAS), and phenolic DESs. SUPRAS is an environmentally friendly, self-assembly, and nanostructured liquid formed by amphiphilic compounds in consecutive phases, including micelles, reverse micelles, and vesica, with types that can be switched by altering the polarity of the continuous phase (Ballesteros-Gómez, Sicilia, & Rubio, 2010). The shape is shown in Fig. 4A. DLLME based on SUPRAS has always been praised for its excellent enrichment efficiency. Qin et al. (2023) developed DLLME based on SUPRAS to extract arctiin, phillyrin, arctigenin, and phillygenin from *Forsythia suspensa* fruit. In this study, reserve micelle-SUPRAS (70 μL) was composed of 5 mmol/L TBAB and n-hexanol. After optimization, outstanding EFs ranging from 6 to 170 were obtained. Moreover, DLLME based on SUPRAS has also shown merits in terms of excellent extraction efficiency. In the study mentioned above, the average recoveries for arctiin, phillyrin, arctigenin, and phillygenin ranged from 96.5 % to 104.8 % (Qin et al., 2023). Phenolic DESs, another kind of green solvents, consist of a hydrogen bond acceptor and a phenolic hydrogen bond donor, capable of forming abundant and strong π-π bonds. Compared with common DESs, phenolic DESs can extract not only polar lignans (e.g., sesamol), but also non-polar lignans (e.g., sesamin and sesamolin) (Liu et al., 2019). One merit of using DLLME based on phenolic DES is its fantastic extraction efficiency. Liu et al. (2019) developed a DLLME method based on phenolic DESs to extract sesamol, sesamin, and sesamolin from sesamol oils. In the study, the phenolic DES consisted of choline chloride and p-cresol. At the best experimental conditions, satisfactory recoveries for sesamol (97.3–113 %), sesamin (102–120 %), and sesamolin (98.4–109 %) were gained. In addition, DLLME based on phenolic DESs has also been praised for saving solvents and time, as well as for its environmentally friendly nature.

DLLME based on solidification of a floating organic drop (SFOD), first put forward by (Khalili Zanjani, Yamini, Shariati, & Jönsson, 2007), makes use of solvents with a melting point (M.P.) approaching normal temperature (10–30 °C) and a density lower than that of water (Geng et al., 2020). The operation procedure of DLLME-SFOD is shown in Fig. 1D. One merit of using DLLME-SFOD is its ability to acquire high enrichment efficiency. Geng et al. (2020) developed a DLLME-SFOD method to extract and purify MG and HK from health foods. In this study, DLLME-SFOD, HF-LPME, OIS-HF-LPME, and DES-HF-LPME were compared, and DLLME-SFOD was selected due to its superior enrichment efficiency. Under optimal conditions, DLLME based on SFOD achieved the highest EFs of 229 and 252, which were 3.23–14 times higher than those of the others. In addition, DLLME-SFOD has also shown advantages in terms of outstanding extraction efficiency, being time-saving, and convenience.

In recent years, energy sources, such as vortex and ultrasonic waves, have been employed as substitutes for dispersants to perform the dispersion function, thereby avoiding the reduction of the distribution coefficient of the analyte in the extractant due to the addition of dispersants (Hu et al., 2019). Vortex-assisted liquid-liquid microextraction (VALLME) can promote the formation of water-in-oil emulsion droplets to act as a dispersant. It is equipped with a specific vortex mixer that ensures a more uniform mixture, quicker initiation, faster mass transfer, and fewer errors (Xue et al., 2021). VALLME has shown advantages in terms of superior enrichment effects. Xue et al. (2021) adopted a VALLME method to extract MG and HK from health foods. In this study, they used tetrabutylammonium chloride-hexanoic acid as the extraction solvent. Under optimum conditions, excellent EFs, 197 for HK and 210 for MG, were attained. Moreover, VALLME has always been praised for its environmental friendliness, in the study mentioned above, the entire pretreatment only cost 300 μL of extraction solvents (Xue et al., 2021).

#### 2.2.4 Summary of different LPME methods

With the progress of research, LPME methods have emerged as innovative techniques for extracting trace amounts of lignans from plant-derived foods. These methods provide enhanced capabilities in terms of enrichment and extraction, while diminishing the usage of solvents and the requirements of time, thereby contributing to environmental sustainability. DI-SDME is acknowledged for its high enrichment factors, with recent advancements mainly focusing on the development of green solvents (e.g., OIS) and materials for protecting droplets (e.g., BT). HF-LPME is particularly suitable for extracting lignans that contain high levels of impurities, offering a cost-effective and efficient method. The developments in HF-LPME revolve around hollow fiber and solvents (e.g., DES and OIS). DLLME enhances the interaction between lignans and adsorbents, thereby cutting down on the experimental time and increasing enrichment ratios. The utilization of energy (e.g., ultrasonic wave and vortex) in place of dispersants and the incorporation of novel solvents (e.g., SUPRAS, SFOD, and phenolic DESs) make the DLLME technique more environmentally friendly, rapid, and economical.

### 2.3 Solid-phase extraction (SPE)

SPE has been developed by integrating liquid-solid extraction columns with liquid chromatography. Compared to LLE, SPE offers greater comfort and ease of automation (Nasiri, Ahmadzadeh, & Amiri, 2020). At present, it is widely applied for purifying and extracting lignans from plant-derived foods due to its excellent properties, such as high selectivity, relative safety, and ease of operation. Commonly used SPE columns for lignans include C_18_ column (ODS) (Zhao, Zhang, Liu, Deng, & Wu, 2017), Oasis hydrophilic-lipophilic balance (HLB) column (Zhang, Chen, Hu, Shen, & Wang, 2018), and diol-bonded phase column (Franco et al., 2014). The C_18_ column, filled with chemically bonded octadecylsilane silica, is a common reversed-phase column. It has many remarkable properties including good versatility, hydrophobicity, cost-effectiveness, and stability. SPE based on C_18_ column has demonstrated advantages in terms of good extraction efficiency. Zhao et al. (2017) developed an SPE method using a C_18_ column to extract silybin and isosilybin from health foods. In this study, methanol was chosen as the elution. At the best conditions, recoveries ranged from 66.7 % to 81.8 %. In addition, SPE based on C_18_ column has always been praised for its simplicity, time-saving nature, and accuracy. Oasis HLB is a bipolar copolymer with both hydrophilic and lipophilic functional groups, which is environmentally friendly, cost-effective, and easy to use. In the purification process, the steps for handling the adsorbent and washing the impurities can be omitted, thus simplifying the procedures (Zhang et al., 2018). One merit of SPE based on Oasis HLB column is its good purification effect. Zhang et al. (2018) developed an SPE method based on Oasis HLB column to extract seven lignans from health foods. In this study, an Oasis PRiME HLB column was chosen as the SPE column, and 1 mL acetonitrile was used as the eluent solvent. Under optimum conditions, the matrix effect ranged from 0.03 to 0.18. In addition, SPE based on Oasis HLB column has also shown advantages in terms of outstanding extraction efficiency, time-saving, and cost-effectiveness.

In recent years, many new SPE materials have been updated to enhance extraction results, such as fibrous silica nanospheres (KCC-1), restricted access materials (RAMs), and molecularly imprinted polymers (MIPs). KCC-1, featuring radially oriented tunnels, is a mesoporous silica microsphere with unique properties, including a large surface area (641 m^2^g^−1^), wide bore diameters (250–450 nm), awesome mechanical strength, and fantastic stability (Polshettiwar, Cha, Zhang, & Basset, 2010). The shape is shown in Fig. 4B. KCC-1 expedites the adsorption of lignans; its external surface presents numerous active sites Error! Bookmark not defined., and its structure helps overcome mass transfer resistance (Polshettiwar et al., 2010). Therefore, SPE based on KCC-1 is highly regarded for its superior extraction effect. Shen et al. (2020) developed an SPE method based on KCC-1 to extract six lignans from *Justicia procumbens* L. (a vegetable). In this study, methanol (3 mL) and acetonitrile (3 mL) were chosen as the activating solvent and the elution solvent, respectively. After optimization, the recoveries of six lignans were 83.4–96.1 %. Moreover, SPE based on KCC-1 has also shown advantages in enhancing the purification effect of lignans. In the study mentioned above, the baseline noise of SPE based on KCC-1 ranged from 50 to 150 cps, which is lower than that of common methods (Shen et al., 2020). RAMs are a class of porous adsorption featuring a dual surface structure, with the external surface modified with hydrophilic groups to repel biomacromolecules (e.g., protein) and the internal surface designed to retain lignans (Fumes, Silva, Andrade, Nazario, & Lanças, 2015). One merit of using SPE based on RAMs is the superior extraction results. Zhou, Hu, Chen, and Zhang (2020) adopted SPE based on RAMs to extract MG and HK. In this study, poly-hydroxyethyl methacrylate (poly-HEMA) was chosen to modify the hydrophilic groups in RAMs. Upon optimal conditions, satisfactory recovery rates of MG (93.0 %) and HK (101.73 %) were obtained in the eluent. Meanwhile, SPE based on RAMs also shows merits in terms of time efficiency. In the experiment mentioned above, the whole extraction only took 10 min (Zhou et al., 2020). MIPs are robust materials that specifically recognize target compounds through a mechanism similar to a “lock and key” (Xu et al., 2024). The formation of MIPs involves adopting target lignans as templates, where polymerizable monomers interact with the lignans' functional monomers. These polymerizable monomers then undergo self-assembly and polymerization under the action of a cross-linking agent, resulting in the formation of MIPs. MIPs exhibit strong selectivity, are cost-effective, and can be reused. One merit of SPE based on MIPs is its high extraction efficiency. Xu et al. (2024) used SPE based on MIPs to extract Schizandrol A, schisantherin A, schizandrin A, and schizandrin B from *S. chinensis* fruit. In this experiment, ethanol-acetic acid (85,15, v,v) was selected as the elution solvent. Under optimal conditions, the recovery rates of the four lignans were 93.13–98.75 %. In addition, SPE based on MIPs also offers the advantages of high specificity, cost savings, and environmental protection.

Monolithic columns represent a new type of porous medium, whose production method differs entirely from that of common packed columns. The monolithic column is characterized by an excellent void ratio (60–80 %), a wide bore diameter, and outstanding chemical stability, facilitating the rapid and stable arrival of lignans at the adsorption site (Svec, 2017). Consequently, SPE based on monolithic column has always been commended for its time-saving nature. Zhou et al. (2020) developed an SPE method based on a monolithic column to extract MG and HK. In this study, styrene (ST) and ethylene glycol dimethacrylate (EDGMA) (2:8, mol/mol) were chosen as substrates for fabricating the monolithic columns, with an ammonia/acetonitrile solution (0.5 %, *v*/v) serving as the adsorbent. After optimization, the entire process took only 10 min. Moreover, SPE based on monolithic column also possesses the merits of good purification efficiency. In the experiment of Zhou et al. mentioned above, a satisfactory average bovine serum protein exclusion rate of 100.59 % was obtained (Zhou et al., 2020).

Microextraction by packed sorbent (MEPS) is a miniaturized SPE technology that simplifies common SPE, offering a rapid and convenient technology suitable for low sample volumes. Requiring no specialized extraction devices, it uses a micro-syringe in place of columns (Yang, Said, & Abdel-Rehim, 2017). MEPS has demonstrated advantages in terms of environmental friendliness. Zhou et al. (2020) developed a MEPS method to extract MG and HK. In this research, a monolithic column was synthesized in a 1000 μL micro-syringe for lignan extraction. After optimization, the process only required 400 μL of organic solvents. In addition. MEPS is characterized by its simplicity, time efficiency, and low cost.

To sum up, SPE is being increasingly acknowledged for its usefulness in extracting multi-polar lignans from complex matrices. Column materials, such as KCC-1, RAMs, and MIPs, represent some of the most common optimization directions in SPE. KCC-1 and RAMs possess a large surface area which accelerates adsorption, thereby increasing the efficiency of SPE. MIPs possess extremely high specificity, thus improving the extraction purity of SPE. In comparison with column materials, monolithic columns present several merits, such as low back pressure, rapid extraction, and excellent stability. MEPS is also a significant advancement in SPE, further enhancing its convenience, efficiency, and environmental sustainability. To conclude, the advancements in SPE have primarily focused on the application of more cost-effective, efficient, and stable column materials, along with the miniaturization of columns.

### 2.4 Dispersive micro solid phase extraction (D-μ-SPE)

D-μ-SPE is an emerging type of solid phase microextraction for extracting lignans. The adsorbed particles are adequately exposed to lignans since they are dispersed within the solvent, thereby resulting in rapid and efficient extraction (Ćirić et al., 2018). One merit of using D-μ-SPE is its high extraction efficiency. Chu et al. (2020) developed a D-μ-SPE method to extract MG and HK from health foods. In this study, 1 mg of silica gel, acting as the adsorbent, was distributed among the samples. After optimization, the recoveries ranged from 99.6 % to 99.7 %. Moreover, D-μ-SPE has also been praised for its environmental friendliness and time efficiency. In the study mentioned above, the process merely required 100 μL of methanol, and the entire extraction was completed within just 2 min (Chu et al., 2020).

**Fig. 2:**
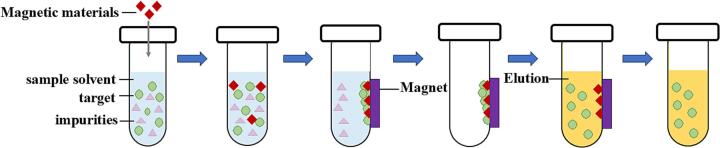
Flow chart of dispersive micro solid phase extraction.

In recent years, novel D-μ-SPE adsorbent materials for lignans have been developed to achieve better results. These novel materials include magnetic graphene oxide (MGO) and polyethylenimine-modified magnetic nanoparticles (PEI-MNPs), which can be attracted to a magnet placed around the container, thereby simplifying the operation. The experimental procedures are shown in Fig. 2. MGO features a honeycombed carbon (sp^2^) structure, and its preparation method is shown in Fig. 4C. The synthesis of MGO is simple, and graphene is inexpensive. Moreover, it possesses unique properties, such as abundant π electrons for efficient π-π interactions with aromatic carbon atoms, a large specific surface area, and significant adsorption capacity, all of which are favorable for capturing trace lignans (Trivedi & Rachchh, 2022; Zhuang, Yu, Ma, & Chen, 2015). One significant advantage of D-μ-SPE based on MGO is its excellent extraction efficiency. Wu et al. (2017) developed a D-μ-SPE method based on MGO to extract sesamol, sesamin, and sesamolin from sesame oil. In this study, hydroxylated Fe_3_O_4_ (0.1 g) was chosen as the magnetic compound to modify GO (0.04 g). After optimization, acceptable recoveries ranging from 85.48 % to 93.78 % were obtained. Moreover, D-μ-SPE based on MGO has also been commended for its time efficiency. In the study by Wu et al. mentioned above, the entire extraction process only took 4.5 min (Wu et al., 2017). PEI-MNPs are metallic inorganic granules modified with PEI. The production of PEI-MNPs is cost-effective and recyclable, and they possess substantial adsorbent capacity and vast specific surface areas (He, Huang, Wang, Zhang, & Li, 2014). Moreover, PEI possesses many amine functional groups capable of forming cation-π interactions and electrostatic adsorptions with the methoxyl and hydroxyl groups of lignans (Zhou et al., 2012). D-μ-SPE based on PEI-MNPs has demonstrated superior extraction efficiency for trace lignans in complicated matrices. Piao et al. (2018) developed a D-μ-SPE method based on PEI-MNPs to extract schisandrol A and angeloylgomisin H from health foods. In this study, methanol was chosen as the eluent, and the pH of the solution was adjusted to 7.4. After optimization, recoveries ranging from 84.1 to 104.4 % were obtained. Moreover, D-μ-SPE based on PEI-MNPs also demonstrates the merits of being time-saving. In the study mentioned above, the process merely consumed 10 min, remarkably shorter than the UAE method (usually more than 30 min) (Piao et al., 2018).

To conclude, D-μ-SPE disperses the adsorbed particles within the sample, thereby enhancing the efficiency of lignan extraction and decreasing solvent consumption. The development of D-μ-SPE mainly centers on the application of novel adsorbent materials, such as magnesium MGO and PEI-MNPs, which make the technique more environmentally friendly.

### 2.5 Supercritical fluid extraction (SFE) and subcritical fluid extraction (SBE)

SFE, an advanced technology, has been widely applied since the 1970s (Jentoft & Gouw, 1970). The most commonly used SFE solvent is carbon dioxide (CO_2_), which has the advantages of being cost-effective, environmentally friendly, and chemically inert. SFE can display both the high solubility characteristic of liquid and the high diffusivity of gas by applying appropriate pressure (P > P_c_) and temperature (T > T_c_) (Saito, 2013). SFE has shown merit in improving the extraction efficiency. Gao, Wu, Cong, Xiao, and Ma (2019) employed SFE to extract twenty-three lignans from *S.chinensis* oil. In this study, SFE and UAE were compared, and SFE was selected due to its higher yield. Under optimal conditions, the total lignans contents were 87.61 ± 1.83 mg/g, which was 80.49 ± 0.99 mg/g higher than that obtained with UAE. Furthermore, SFE has always been commended for its time efficiency, cost-effectiveness, and absence of environmental pollution. Recently, ameliorants (e.g., methanol and ethanol) have been added to CO_2_ to improve the polarity for extracting highly polar lignans, since supercritical CO_2_ alone does not have a significant impact on highly polar lignans like pinoresinol, phyllanthin, and niranthin. Pereira et al. (2017) developed an SFE method to extract phyllanthin and niranthin from *Phyllanthus amarus* Schum & Thonn. In this study, SFE1 (CO_2_) and SFE2 (CO_2_ + 50 % ethanol-water) were contrasted, and SFE2 was chosen due to its higher yield. Under optimum conditions, the yields of two lignans using SFE2 were nearly double those of SFE1. However, the equipment required for SFE is relatively costly (Khoddami, Wilkes, & Roberts, 2013).

SBE is also a green technology that has demonstrated significant potential in sample pretreatment, owing to its numerous advantages, including rapid mass transfer and diffusivity, low operating pressure and energy consumption, environmental sustainability, and cost-effectiveness (Plaza & Turner, 2015; Zhang, Li, & Hu, 2024). Currently, solvents that can be used for lignan extraction include water, propane, n-butane, and dimethyl ether (Qin et al., 2024; Wang, Zhang, Chi, & Chen, 2018; Zhang et al., 2024). Their subcritical state can be maintained by controlling the temperature (boiling point<T<T_c_) and pressure (vapor pressure<P<P_c_) (Teo, Tan, Yong, Hew, & Ong, 2010; Zhang et al., 2024). One merit of SBE is its splendid extraction efficiency. Wang et al. (2018) developed SBE based on water to extract podophyllotoxin from health foods. In this study, the temperature and the pressure were set to 180 °C and 4 MPa, respectively. Under optimal conditions, satisfactory recoveries (83.80 %) were obtained. In addition, SBE has always been praised for its low cost, low energy consumption, and ease of industrial production.

To sum up, SFE is one of the advanced methods. CO_2_, which is non-toxic, can be easily recovered in its gaseous form by adjusting the pressure and the temperature, thereby possessing great potential for environmental protection. However, the requirement for advanced equipment is a significant limitation. SBE is an emerging technique that has not been as widely adopted as SFE. Nonetheless, it holds promise for lignan extraction because of its lower costs and reduced energy consumption. Additionally, the use of subcritical water also contributes to environmental protection.

### 2.6 Matrix solid-phase dispersion (MSPD)

**Fig. 3:**
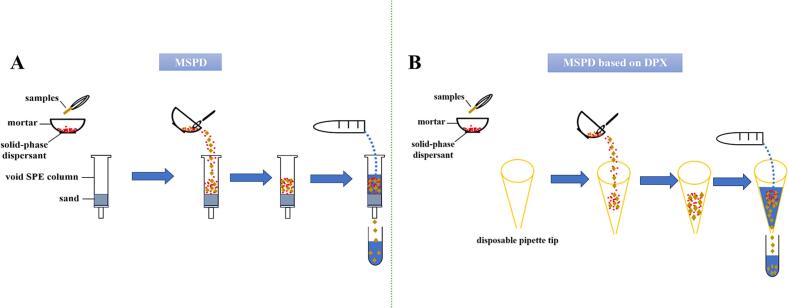
Flow chart of 2 matrix solid-phase dispersion technologies.

MSPD, initially proposed by Barker, Long, and Short (1989), is a rapid method for extracting compounds from solid, semi-solid, or viscous samples (See Table 2) (Chen, Ji, Chen, & Li, 2019; Chu et al., 2017; Chu, Jiang, Mao, & Yan, 2021; Du, Wang, Xiao, & Zhu, 2023; Piao et al., 2018; Qian et al., 2022; Song et al., 2022; Wang, Zhu, Du, & Li, 2023; Wu et al., 2017). MSPD is a simple, cost-effective, and convenient technique, because it combines extraction and purification in one step, omitting the need for filtration, concentration, and other elaborate processes (Tu & Chen, 2018). The procedures of MSPD are shown in Fig. 3A. One significant merit of MSPD is its improvement in extraction efficiency. Chen et al. (2019) developed an MSPD method to extract nine lignans from health foods. In this research, MSPD, soxhlet extraction (SE), and UAE were compared, and MSPD was selected due to its superior performance. After optimization, the recoveries ranged from 93.47 % to 103.52 %, and the total extraction yield of MSPD (4.919 mg/g) was higher than that of SE (4.812 mg/g) and UAE (4.691 mg/g). In addition, MSPD has also shown advantages in terms of being time-saving and environmentally friendly.

**Table 2:** MSPD methods for lignans in food.

Sample	Target analytes	SPME methods	Extraction solvent	SPME process	SPME results	Reference
Health food	Magnolol; Honokiol	Matrix solid-phase dispersion	70 % ethanol	① Put the sample powder (200 mg) and dispersant (200 mg) into an agate mortar ② Grind for 0.5 min ③ Transfer the mixture (50 mg) into a glass tube ④ Add 5 mL 70 % ethanol ⑤ Extract the sample for 1.0 min by ultrasonic extraction (33 kHz, 250 W) ⑥ Add 70 % ethanol solution to compensate the lost weight ⑦ Filter the solution by 0.45 μm membrane	Recoveries: 98.08 %–99.28 %	(Qian et al., 2022)
Health food	Magnolol; Honokiol	Matrix solid-phase dispersion	Methanol	① Grind health food and passing through 80 meshes ② Grind adsorbent (3 mg) and sample powder (6 mg) in an agate mortar for 130 s ③ Transfer the mixtures to a pipette tip using degreased cotton on both ends ④ Use various 200 μL of eluent (methanol, acetonitrile, and ethanol) to elute the cartridges ⑤ Collect the eluent in a 1.5 mL centrifuge tube ⑥ Use eluent to make up to volume ⑦ Centrifuge the supernatant of the eluent at 13,000 rpm for 5 min	Recoveries: 92.16 %–97.10 %	(Chu et al., 2021)
Health foods	9 lignans	Matrix solid-phase dispersion	Methanol	① Place sample (∼0.2 g) and silica gel (0.4 g) into an agate mortar ② Blend and grind (∼3 min) them with a pestle ③ Introduce 0.4 g the mixture into a 10 mL syringe prefilled with a layer of absorbent cotton ④ Use methanol to elute the packed syringe ⑤ Collect the eluent in a volumetric flask (5 mL) ⑥ Use methanol to make up to volume ⑦ Filter the sample solution through a 0.45 μm membrane filter	Recoveries: 93.49 %–103.52 %	(Chen et al., 2019)
Health foods	32 lignans	Magnetic dispersive micro-solid-phase extraction	Methanol	① Use methanol (3 × 20 mL, 20 min each) to extract the dried sample homogeneous powder (2 g) in an ultrasonic bath ② Filtrate and concentrate the combined extracts under reduced pressure ③ Dissolve the resulting extract powder in 100 mL of phosphate buffer (67 mM, pH 7.4) ④ Centrifuge at 10000 rpm for 10 min ⑤ Collect the supernatant ⑥ Mix (20 mg/mL in PB solution) the supernatant (50 μL) with PEI-MNPs (10 mg/mL, 50 μL) ⑦ Incubate for 10 min ⑧ Add 0.4 T magnetic field to separate the PEI-MNPs ⑨ Wash the incubated PEI-MNPs (three times) with 100 μL of PB solution ⑩ Use methanol (100 μL) to desorb the particles and utilize magnetic field to separate PEI-MNPs	Recoveries for schisandrol A and angeloylgomisin H: 84.1–104.4 %	(Piao et al., 2018)
*S. chinensis* fruits	Schisandrin; Gomisin A; Schisantherin A; γ-schisandrin; Deoxyschisandrin	Matrix solid-phase dispersion	Methanol	① Put the sample powder (25 mg) and TS-1 (50 mg) into an agate mortar ② Blend and grind (150 s) them with a pestle ③ Transfer the mixture to an SPE cartridge with a sieve plate at the bottom ④ Put the other sieve plate on top of the blend ⑤ Use methanol (500 μL) to elute the cartridge ⑥ Collect the eluent ⑦ Use extraction solvent to make up to volume ⑧ Centrifuge the eluent at 13,000 rpm for 5 min before injection	Recoveries: 99.3 %–102.2 %	(Chu et al., 2017)
*S. chinensis* fruits	Schisandrol A; Schisandrol B; Schisantherin A; γ -Schisandrin; Schisandrin C Deoxyschisandrin	Matrix solid-phase dispersion	Ethanol	① Put the sample (2.0 g) and neutral alumina (4.0 g) into an agate mortar ② Blend together using an agate pestle ③ Place the mixture and absolute ethanol (25 mL) into a conical flask ④ Shake (2.5 h, 150 rpm) at 40 °C in a desktop thermostatic oscillator ⑤ Concentrate the desorbed solutions in a rotary evaporator ⑥ Dry (until constant weight) in a vacuum drying oven at 45 °C ⑦ Dissolve the drying total by a certain amount of absolute ethanol ⑧ Transfer to a 250 mL volumetric flask ⑨ Use extraction solvent to make up to volume ⑩ Filter through a 0.22 μm filter membrane before HPLC analysis	Recoveries: 93.33 %–102.22 %	(Song et al., 2022)
*S. chinensis* fruit	Schisandrol A; Schisandrol B; Schizandrin B; Schizandrin C; Deoxyschizandrin	Matrix solid-phase dispersion	Methanol	① Place 0.25 g sample and 0.75 g Xion (adsorbent) into an agate mortar ② Blend the mixture using an agate pestle for 3–5 min ③ Introduce the mixture into a 12 mL glass syringe prefilled with a layer of absorbent cotton at the bottom ④ Place a second layer of absorbent cotton by slight compression using a syringe piston ⑤ Rinse the packed syringe with 15 mL of methanol ⑥ Collect the eluent in a 25 mL of volumetric flask ⑦ Use methanol to make up to volume ⑧ Filter the sample solution through a 0.45 μm filter membrane	Recoveries: 92.20–111.2 %	(Du, Wang, Xiao, & Zhu, 2022)
*S. chinensis* fruit	10 lignans	Matrix solid-phase dispersion	85 % methanol-water solution	① Place sample powder (200 mg) and diol-functionalized silica (adsorbent) (800 mg) into an agate mortar ② Blend the mixture using an agate pestle for 5 min ③ Introduce the mixture into a 12 mL polypropylene SPE cartridge with a layer of absorbent cotton at the bottom ④ Place a second layer of absorbent cotton by slight compression using a syringe piston ⑤ Rinse the packed syringe with 10 mL of 85 % methanol-water solution ⑥ Collect the eluent in a volumetric flask ⑦ Use methanol to make up for volume ⑧ Filter the sample solution through a 0.22 μm filter membrane	The 10 lignans were effectively extracted	(Wang et al., 2023)
Sesame oil	Sesamol; Sesamin Sesamolin	Magnetic dispersive micro-solid-phase extraction	Dichloromethane	① Place sesame oil (0.1 g) and dichloromethane (4 mL) into a test tube ② Vortex and dissolve the mixture ③ Add 1.4 mg magnetic graphene oxide in the tube ④ Vortex for 3 min ⑤ Place an external magnetic field at the bottom of the tube ⑥ Remove the supernatant by the magnetic field ⑦ Clean up by 1 mL dichloromethane solution ⑧ Add 1.7 mL methanol into the remaining precipitate ⑨ Vortex-shaking for 1.5 min for desorption ⑩ Separate them by the external magnetic field and collect the supernatant for HPLC analysis	Average recoveries: 81.73 %–93.52 %	(Wu et al., 2017)

**Fig. 4:**
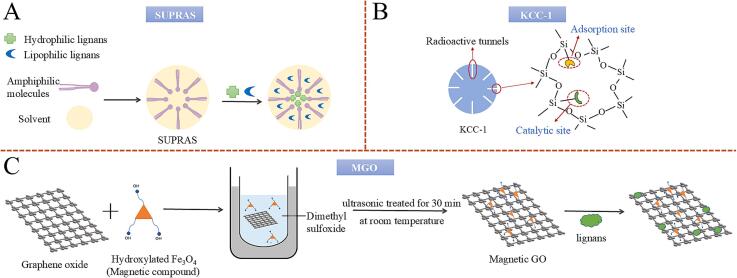
Preparation method of 3 novel materials.

Recently, many new MSPD adsorbent materials have been the focus of innovation. Molecular sieve, a novel type of adsorbent, is broadly applied in MSPD. It is an aluminosilicate with a tetrahedral 3D interaction network composed of silica dioxide and alumina (Anunziata, Beltramone, Martínez, & Belon, 2007). It is cost-effective, readily available, straightforward to use, and environmentally friendly. One merit of MSPD based on molecular sieve is its excellent extraction efficiency. Chu et al. (2021) developed an MSPD method based on molecular sieve SBS-3 to extract HK and MG from health foods. In this study, 6 mg of sample powder and 3 mg of TS-1 were ground for 130 s in an agate mortar. After optimization, satisfactory recoveries ranging from 92.16 % to 97.10 % were obtained. Furthermore, MSPD based on molecular sieve has also shown advantages in environmental friendliness. Chu et al. (2017) developed an MSPD based on molecular sieve TS-1 to extract schisandrin, gomisin A, schsantherin, deoxyschisandrin, and γ-schisandrin from *S.chinensis* fruit. In this study, the mass ratio of sample to dispersant was 1:2. Under the optimal conditions, the MSPD based on TS-1 method used merely 0.05 g of TS-1 and 0.5 mL of methanol for elution.

The container is another area of active innovation. Now, disposable pipette tip (DPX) and conical flask have been used as alternatives to glass syringes. DPX are open at both ends, and elution can be accelerated by squeezing the top of the DPX near the end of the process (Chu et al., 2021). The procedures of MSPD based on DPX are shown in Fig. 3B. One merit of MSPD based on DPX is its time-saving nature. (Chu et al., 2021) utilized MSPD with DPX to extract MG and HK from health foods. In this study, 6 mg of SBA-3 was selected as the adsorbent, and 200 μL of methanol, ethanol, and acetonitrile were chosen as eluents. After optimization, the total process cost 9 min, including 130 s for grounding. In addition, MSPD based on DPX has demonstrated merits such as simplicity in operation, low cost, high extraction efficiency, and effective purification. Conical flask can also serve as a container. Compared to glass syringe, conical flask have a large capacity and can be placed on a desktop thermostatic oscillator for extraction and desorption (Song et al., 2022). One merit of MSPD based on a conical flask is the pronouncedly increased extraction efficiency. Song et al. (2022) developed an MSPD method based on a conical flask to extract six lignans from *S.chinensis* fruits. In this study, MSPD, SE, MAE, UAE, heat reflux extraction, and smashing tissue extraction were compared, with MSPD demonstrating the highest recoveries. At the optimal condition, the total yield of six lignans was 16.99 ± 0.33 × 10^3^ mg/kg, which was 0.05–4.53 mg/kg higher than that of the other five methods. Moreover, MSPD based on a conical flask has always been praised for its speed, convenience, and cost-effectiveness.

To conclude, MSPD is a superior method that improves upon SPE by simplifying the complex processes associated with extracting solid and semi-solid samples, offering a particular advantage for intricate samples. The advancements in MSPD are mainly centered on optimizing the adsorbent materials (e.g., molecular sieves), and the choice of containers (e.g., DPX), which contribute to the miniaturization of MSPD. This not only accelerates the extraction process but also saves both time and resources.

## 3 Conclusion and future prospect

Lignans, plant-derived compounds, exhibit variability in content and structure among numerous foods and health products. However, an excess addition of lignans can result in adverse reactions, such as endocrine disorders and breast cancer. Therefore, it is of crucial importance to develop sensitive, accurate, rapid, and environmentally friendly pretreatment methods for lignan extraction in order to achieve precise quantification. Effective pretreatment techniques are capable of enhancing extraction efficiency, minimizing solvent usage, reducing experimental time, cutting down costs, alleviating environmental pollution, and enhancing the enrichment of trace lignans.

In this review, we have carried out a comparison of various pretreatment methods used for the extraction and purification of lignans (See Table S2) (An et al., 2021; Angeloni et al., 2020; Bhatt, Sharma, Kumar, Sharma, & Singh, 2017; Cea Paze et al., 2019; Chen et al., 2024; Chu et al., 2020; Gao et al., 2019; Geng et al., 2020; Guan et al., 2018; Guo et al., 2013; Li, Chen, et al., 2018a; Liu et al., 2019; Michailidis, Angelis, Aligiannis, Mitakou, & Skaltsounis, 2019; Pereira et al., 2017; Piao et al., 2018; Qin et al., 2023; Shen et al., 2020; Wang et al., 2018; Wang et al., 2019; Wu et al., 2017; Wu et al., 2021; Xia et al., 2020; Xu et al., 2024; Xue et al., 2019; Xue et al., 2021; Zhao et al., 2017; Zhou et al., 2020), while also assessing their respective advantages and disadvantages (See Table 3) (Hu, Zhang, Zhou, Liu, & Feng, 2022; Li et al., 2021; Li & Row, 2019; Nasiri et al., 2020; Patyra et al., 2022; Zhang, Xing, Yang, Fei, & Liu, 2022). Solvent extraction is one of the commonly adopted methods for lignan extraction on account of their simplicity. However, this method requires substantial amounts of toxic solvents and time. Therefore, many green solvents (e.g., ILs, HILs, and DES) and assisted techniques (e.g., UAE, MAE, EAE, and PLE) are applied in solvent extraction to reduce solvent usage and enhance extraction efficiency, thereby making the process more environmentally friendly. LLE has gained recognition for analyzing lignans due to its simplicity. In contrast to LLE, SPE offers greater applicability for the analysis of multi-polar lignans in complex matrices. Column materials (e.g., KCC-1, RAMs, and MIPs) that are lower in cost, higher in efficiency and stability, as well as monolithic column and column miniaturization, are the focuses of SPE research, making SPE more efficient, convenient, and green. With the development of technology, microextraction techniques have been developed, such as DI-SDME, HF-LPME, DLLME, and D-μ-SPE, which enhance extraction efficiency, reduce time and solvent consumption, and comply with the principles of green chemistry. DI-SDME has high enrichment ratios, and its updates mainly focus on developing green solvents (e.g., OIS) and droplet protection materials (e.g., BT). HF-LPME offers better solvent protection than DI-SDME and is ideal for analyzing lignans in samples with numerous impurities. The development of HF-LPME concentrates on optimizing hollow fiber and solvent (e.g., DES and OIS). DLLME enhances the contact areas between lignans and adsorbents and shortens the analysis time by using energy sources to replace dispersion (e.g., ultrasonic wave and vortex) and novel solvents (e.g., SUPRAS and phenolic DESs), thereby making DLLME greener, faster, and cheaper. SFOD-DLLME achieves highly enriched results with short extraction time and easy operation, but it requires solvents with melting points close to room temperature and densities lower than that of water. D-μ-SPE, which originates from SPE, has shown merits in superior extraction efficiency, solvent savings, and time efficiency, whose updates mainly focus on novel adsorbing materials (e.g., MGO and PEI-MNPs). SFE and SBE are both advanced techniques that can achieve supercritical and subcritical states of solvents by adjusting temperature and pressure, respectively. Their advantages include environmental protection, ease of solvent recovery, and reduced resource waste. Currently, SBE is not as vast employment as SFE. However, SBE still holds great promise for lignan extraction because it can use more common solvents like water and does not require the expensive equipment needed for SFE. Furthermore, MSPD facilitates the extraction and purification of lignans from solid or semi-solid samples. The utilization of novel extraction solvents (e.g., OIS, DES, SUPRAS, and SFOD) and adsorbent materials (e.g., KCC-1, RAM, BT, MGO, PEI-MNPs, molecular sieve, and DPX) makes these novel techniques more cost-effective and effective.

**Table 3:** The advantages and disadvantages of methods used for extracting lignans from food samples. (Hu, Zhang, Zhou, Liu, & Feng, 2022; Li et al., 2021; Li & Row, 2019; Nasiri et al., 2020; Patyra et al., 2022; Zhang, Xing, et al., 2022).

Pretreatment methods		Advantages	Disadvantages
Solvent extraction		① Wide applicability ② Low cost ③ Simple operation ④ Efficient extraction	① Time-consuming extraction process ② Low selectivity ③ Potential environmental impact ④ High toxic solvent consumption
UAE		① Shorten extraction times ② Improve extraction efficiency ③ Reduce solvent consumption ④ Save the energy	① Possible structure changes of compounds ② Complex operating conditions ③ High temperature affects extraction efficiency
EAE		① Shorten extraction times ② Improve extraction efficiency ③ Reduce solvent consumption ④ Save the energy	① Risk of thermal degradation ② Complex operating conditions ③ Specialized equipment required ④ Safety concerns with microwaves
MAE		① Specificity in extraction ② Improve extraction efficiency ③ Milder operating conditions ④ Reduced environmental impact	① Higher cost of enzymes ② Limited by enzyme activity and stability ③ Possible enzyme inhibition by matrix components
PLE		① High extraction efficiency ② Short extraction times ③ Reduced solvent consumption ④ Automation capability	① High initial equipment cost ② Complexity in operation ③ Potential for high pressure safety issues.
LLE		① Simple and conventional method ② Low cost ③ No special equipment needed ④ Applicable to a wide range of compounds	① High toxic solvent consumption ② Labor-intensive and time-consuming procedure ③ Multi-step process ④ Tendency for emulsion formation
LPME	DI-SDME	① Low solvent consumption ② High enrichment factor ③ Simple operation ④ Extract trace lignans	① Susceptible to droplet loss; ② Limited to small sample volumes ③ Requires careful handling
	HF-LPME	① Low solvent consumption ② High enrichment factor ③ Wide pH ranges ④ Ease of operation	① Potential for fiber clogging ② Requires specialized equipment ③ Possible carry-over effects
	DLLME	① Low solvent consumption ② High enrichment factor ③ Rapid extraction process ④ Protect the environment	① Possible emulsion formation ② Requires centrifugation or filtration ③ Requires disperser solvent
SPE	SPE	① High purification eff ② Ease of automation and minimization ③ Wide range of sorbent options ④ Compatibility with various detectors	① Relatively larger solvent consumption ② Relatively larger extraction time ③ The blockage of cartridges
	MEPS	① High selectivity and recovery ② Rapid and easy operation ③ Low sorbent and solvent usage ④ No specialized extracted devices ⑤ Environmentally friendly	① The limited number of available sorbents ② Lower sample capacity ③ Possibility of packed sorbent getting clogged
SPME	D-μ-SPE	① High enrichment factor ② Simple and rapid operation ③ Low solvent usage ④ Avoiding costly SPE column or SPME fiber.	① Difficulty of the sorbent separation from the sample solution. ② Complex operation steps
SFE		① High extraction efficiency ② Simple and rapid operation ③ Low solvent usage ④ Rapid extraction process ⑤ Environmental friendliness	① High equipment cost ② Limited to certain compound polarities ③ High pressure requirements
SWE		① Environmentally benign solvent ② Rapid mass transfer and diffusivity ③ High extraction efficiency ④ Rapid extraction process ⑤ Low cost	① Possible thermal degradation of compounds ② Specialized equipment required
MSPD		① High extraction efficiency ② Reduces sample handling ③ Suitable for solid and semi-solid samples. ④ Simplifies extraction and purification	① Limited to certain sorbents ② Possible loss of sample

In the future, the focus of lignan pretreatment development will lie in the following aspects: (1) The development and utilization of more environmentally friendly solvents and adsorbent materials, which will be beneficial for reducing experimental costs, shortening experimental times, enhancing extraction efficiency, and protecting the environment. (2) The employment of automated pretreatment technologies. Some methods like SPE possess great potential for automation. Automated pretreatment technologies allow for the automatic execution of extraction and purification processes, minimizing manual intervention and experimental errors, thereby enhancing accuracy. (3) The development of miniaturized techniques. Miniaturized techniques, originating from LLE and SPE, can achieve faster extraction, better extraction performance, and lower sample consumption, which is beneficial for the extraction of trace lignans from complex matrices. Overcoming these challenges will not only promote the extraction and purification of lignans but also make contributions to the broader fields of food chemistry and nutraceutical development.

## CRediT authorship contribution statement

**Yu-tong Yang:** Writing – original draft. **Yuan Zhang:** Writing – original draft, Conceptualization. **Yu Bian:** Writing – original draft. **Juan Zhu:** Writing – review & editing. **Xue-song Feng:** Conceptualization.

## Declaration of competing interest

The authors declare that no competing financial interests or personal relationships that could have influence the work reported in this paper.

## Data Availability

No data was used for the research described in the article.

## Appendix A Supplementary data

**Fig. S1.** Four monomers of lignans. **Fig. S2.** Six secondary structures of lignans Image 1

## References

[bb0005] Abranches D.O., Martins M.A.R., Silva L.P., Schaeffer N., Pinho S.P., Coutinho J.A.P. (2019). Phenolic hydrogen bond donors in the formation of non-ionic deep eutectic solvents: The quest for type V DES. Chemical Communications (Cambridge, England).

[bb0010] An J., He C., Guo C., Dong Z. (2021). Application of hollow fiber centrifugal ultrafiltrate purification as the pretreatment technology for traditional Chinese medicine: Its application for analysis of honokiol and magnolol in TCM preparations containing cortex Magnoliae officinalis. Ann Palliat Med.

[bb0015] Angeloni S., Navarini L., Khamitova G., Maggi F., Sagratini G., Vittori S., Caprioli G. (2020). A new analytical method for the simultaneous quantification of isoflavones and lignans in 25 green coffee samples by HPLC-MS/MS. Food Chemistry.

[bb0020] Anunziata O.A., Beltramone A.R., Martínez M.L., Belon L.L. (2007). Synthesis and characterization of SBA-3, SBA-15, and SBA-1 nanostructured catalytic materials. Journal of Colloid and Interface Science.

[bb0025] Ballesteros-Gómez A., Sicilia M.D., Rubio S. (2010). Supramolecular solvents in the extraction of organic compounds. A review. Analytica Chimica Acta.

[bb0030] Barker S.A., Long A.R., Short C.R. (1989). Isolation of drug residues from tissues by solid phase dispersion. Journal of Chromatography A.

[bb0035] Bess E.N., Bisanz J.E., Yarza F., Bustion A., Rich B.E., Li X., Turnbaugh P.J. (2020). Genetic basis for the cooperative bioactivation of plant lignans by Eggerthella lenta and other human gut bacteria. Nature Microbiology.

[bb0040] Bhatt V., Sharma S., Kumar N., Sharma U., Singh B. (2017). Simultaneous quantification and identification of flavonoids, lignans, coumarin and amides in leaves of Zanthoxylum armatum using UPLC-DAD-ESI-QTOF-MS/MS. Journal of Pharmaceutical and Biomedical Analysis.

[bb0045] Bişgin A.T. (2023). The novel extraordinary separation and preconcentration approach for Cd^2+^: “Hydrophobic immiscible chelating fluid” based micro-extraction. Journal of Molecular Liquids.

[bb0050] Bişgin A.T. (2024). Effective and selective type V deep eutectic solvent based microextraction of E127 in foodstuffs and drugs. Journal of Food Composition and Analysis.

[bb0055] Caprioli G., Boarelli M.C., Ricciutelli M., Sagratini G., Fiorini D. (2019). Micro-scaled quantitative method to analyze olive oil polyphenols. Food Analytical Methods.

[bb0060] Cea Paze I., Lozano-Sanchez J., Borras-Linares I., Nunez H., Robert P., Segura-Carretero A. (2019). Obtaining an extract Rich in phenolic compounds from olive pomace by pressurized liquid extraction. Molecules.

[bb0065] Chen B., Liu S., Zhi H., Huang J., Song H., Fan J., Liu Z. (2024). Microwave-assisted deep eutectic solvent extraction of five lignans from Schisandra chinensis. Separation Science Plus.

[bb0070] Chen H., Ji T., Chen J., Li X. (2019). Matrix solid-phase dispersion combined with HPLC-DAD for simultaneous determination of nine Lignans in Saururus chinensis. Journal of Chromatographic Science.

[bb0075] Chen J., Chen Y., Tian J., Ge H., Liang X., Xiao J., Lin H. (2018). Simultaneous determination of four sesame lignans and conversion in Monascus aged vinegar using HPLC method. Food Chemistry.

[bb0080] Chu C., Jiang L., Mao H., Yan J. (2021). A simple and environmentally-friendly method by pipette-tip matrix solid-phase dispersion microextraction coupled with high-performance liquid chromatography for the simultaneous determination of lignans and terpenes. Sustainable Chemistry and Pharmacy.

[bb0085] Chu C., Li J., Wang S., Weng L., Jiang L., Zhang H., Yan J. (2020). A simple and sensitive dispersive Micro-solid-phase extraction coupled with high-performance liquid chromatography for quantification of Honokiol and Magnolol in complex matrices. Journal of AOAC International.

[bb0090] Chu C., Wei M., Wang S., Zheng L., He Z., Cao J., Yan J. (2017). Micro-matrix solid-phase dispersion coupled with MEEKC for quantitative analysis of lignans in Schisandrae Chinensis Fructus using molecular sieve TS-1 as a sorbent. Journal of Chromatography. B, Analytical Technologies in the Biomedical and Life Sciences.

[bb0095] Ćirić S., Mitić V., Jovanović S., Ilić M., Nikolić J., Stojanović G., Stankov Jovanović V. (2018). Dispersive micro-solid phase extraction of 16 priority polycyclic aromatic hydrocarbons from water by using thermally treated clinoptilolite, and their quantification by GC-MS. Microchimica Acta.

[bb0100] Du X.-X., Wang Y.-P., Xiao W., Zhu J.-B. (2023). Simultaneous determination of five lignans from Schisandra chinensis by matrix solid-phase dispersion extraction-high performance liquid chromatography. Se pu = Chinese Journal of Chromatography.

[bb0105] Fakhari S., Sharifi M., De Michele R., Ghanati F., Safaie N., Sadeghnezhad E. (2019). Hydrogen sulfide directs metabolic flux towards the lignan biosynthesis in Linum album hairy roots. Plant Physiology and Biochemistry.

[bb0110] Farajzadeh M.A., Sorouraddin S.M., Mogaddam M.R.A. (2014). Liquid phase microextraction of pesticides: A review on current methods. Microchimica Acta.

[bb0115] Ferraz R., Branco L.C., Prudêncio C., Noronha J.P., Petrovski Ž. (2011). Ionic liquids as active pharmaceutical ingredients. ChemMedChem.

[bb0120] Franco M.N., Galeano-Diaz T., Lopez O., Fernandez-Bolanos J.G., Sanchez J., De Miguel C., Martin-Vertedor D. (2014). Phenolic compounds and antioxidant capacity of virgin olive oil. Food Chemistry.

[bb0125] Fumes B.H., Silva M.R., Andrade F.N., Nazario C.E.D., Lanças F.M. (2015). Recent advances and future trends in new materials for sample preparation. TrAC Trends in Analytical Chemistry.

[bb0130] Gao Y., Fan M., Cheng X., Liu X., Yang H., Ma W., Li L. (2024). Deep eutectic solvent: Synthesis, classification, properties and application in macromolecular substances. International Journal of Biological Macromolecules.

[bb0135] Gao Y., Gu C., Wang K., Wang H., Ruan K., Xu Z., Feng Y. (2018). The effects of hypoglycemia and weight loss of total lignans from Fructus Arctii in KKAy mice and its mechanisms of the activity. Phytotherapy Research.

[bb0140] Gao Y., Wu S., Cong R., Xiao J., Ma F. (2019). Characterization of lignans in Schisandra chinensis oil with a single analysis process by UPLC-Q/TOF-MS. Chemistry and Physics of Lipids.

[bb0145] Geng X., Chen X., Li Z., Bai X., Hu S. (2020). Application of solidified floating double-solvent dispersive liquid-phase microextraction for the analysis of the main active components in Zicao Chengqi decoction. SN. Applied Sciences.

[bb0150] Guan L., Luo Q., Liang N., Yu W. (2018). Determination of Lignans in Schisandra sphenanthera and Schisandra chinensis using ionic liquid-based ultrasonic-assisted extraction and high-performance liquid chromatography. Chemical Research in Chinese Universities.

[bb0155] Guo H., Pang X., Zhang W., Jiang W., Pang X. (2013). Dissolution determination of five components in Huoxiang Zhengqi tablets using partitioned dispersive liquid-liquid microextraction combined with HPLC-UV. Analytical Methods.

[bb0160] Han D., Row K.H. (2012). Trends in liquid-phase microextraction, and its application to environmental and biological samples. Microchimica Acta.

[bb0165] He J., Huang M., Wang D., Zhang Z., Li G. (2014). Magnetic separation techniques in sample preparation for biological analysis: A review. Journal of Pharmaceutical and Biomedical Analysis.

[bb0175] Hu C., Zhang Y., Zhou Y., Liu Z.F., Feng X.S. (2022). Unsymmetrical dimethylhydrazine and related compounds in the environment: Recent updates on pretreatment, analysis, and removal techniques. Journal of Hazardous Materials.

[bb0180] Hu S., Chen X., Wang R.-Q., Yang L., Bai X.-H. (2019). Natural product applications of liquid-phase microextraction. TrAC Trends in Analytical Chemistry.

[bb0185] Imai T., Nomura M., Fukushima K. (2006). Evidence for involvement of the phenylpropanoid pathway in the biosynthesis of the norlignan agatharesinol. Journal of Plant Physiology.

[bb0190] Jafernik K., Ekiert H., Szopa A. (2023). Schisandra henryi-a rare species with high medicinal potential. Molecules.

[bb0195] Jain A., Verma K.K., Poole C.F. (2020). Liquid-Phase Extraction.

[bb0200] Jeannot M.A., Cantwell F.F. (1996). Solvent microextraction into a single drop. Analytical Chemistry.

[bb0205] Jentoft R.E., Gouw T.H. (1970). Pressure-programmed supercritical fluid chromatography of wide molecular weight range mixtures*. Journal of Chromatographic Science.

[bb0210] Ji B., Xia B., Fu X., Lei S., Ye Y., Zhou Y. (2018). Low-cost and convenient ballpoint tip-protected liquid-phase microextraction for sensitive analysis of organic molecules in water samples. Analytica Chimica Acta.

[bb0215] Jiang R.W., Jaroch K., Pawliszyn J. (2023). Solid-phase microextraction of endogenous metabolites from intact tissue validated using a biocrates standard reference method kit. Journal of Pharmaceutical Analysis.

[bb0220] Kaçanbüre D., Bişgin A.T. (2025). Selective microextraction of erythrosine (E127) in foodstuffs using a new generation high-density type-V deep eutectic solvent. Food Chemistry.

[bb0225] Khalili Zanjani M.R., Yamini Y., Shariati S., Jönsson J.Å. (2007). A new liquid-phase microextraction method based on solidification of floating organic drop. Analytica Chimica Acta.

[bb0230] Khoddami A., Wilkes M.A., Roberts T.H. (2013). Techniques for analysis of plant phenolic compounds. Molecules.

[bb0235] Kim S.J., Shin H., Cheon S.M., Ko S.M., Ham S.H., Kwon Y.D., Cho H.Y. (2017). A sensitive UHPLC-MS/MS method for the simultaneous quantification of three lignans in human plasma and its application to a pharmacokinetic study. Journal of Separation Science.

[bb0240] Landete J.M. (2012). Plant and mammalian lignans: A review of source, intake, metabolism, intestinal bacteria and health. Food Research International.

[bb0245] Leong M.-I., Fuh M.-R., Huang S.-D. (2014). Beyond dispersive liquid–liquid microextraction. Journal of Chromatography A.

[bb0250] Li G., Row K.H. (2019). Utilization of deep eutectic solvents in dispersive liquid-liquid micro-extraction. TrAC Trends in Analytical Chemistry.

[bb0255] Li M., Chen X., Hu S., Wang R., Peng X., Bai X. (2018). Determination of blood concentrations of main active compounds in Zi-Cao-Cheng-qi decoction and their total plasma protein binding rates based on hollow fiber liquid phase microextraction coupled with high performance liquid chromatography. Journal of Chromatography. B, Analytical Technologies in the Biomedical and Life Sciences.

[bb0260] Li M., Chen X., Hu S., Wang R., Peng X., Bai X. (2018). Determination of blood concentrations of main active compounds in Zi-Cao-Cheng-qi decoction and their total plasma protein binding rates based on hollow fiber liquid phase microextraction coupled with high performance liquid chromatography. Journal of Chromatography B.

[bb0265] Li M., Hu S., Chen X., Wang R., Bai X. (2018). Research on major antitumor active components in Zi-Cao-Cheng-qi decoction based on hollow fiber cell fishing with high performance liquid chromatography. Journal of Pharmaceutical and Biomedical Analysis.

[bb0270] Li M.M., Hu S., Chen X., Bai X.H. (2017). Development of a novel hollow-fiber liquid-phase microextraction based on oil-in-salt and its comparison with conventional one. Journal of Separation Science.

[bb0275] Li N., Zhang T., Chen G., Xu J., Ouyang G., Zhu F. (2021). Recent advances in sample preparation techniques for quantitative detection of pharmaceuticals in biological samples. TrAC Trends in Analytical Chemistry.

[bb0280] Liu K., Song Y., Liu Y., Peng M., Li H., Li X., Su D. (2017). An integrated strategy using UPLC-QTOF-MS(E) and UPLC-QTOF-MRM (enhanced target) for pharmacokinetics study of wine processed Schisandra Chinensis fructus in rats. Journal of Pharmaceutical and Biomedical Analysis.

[bb0285] Liu W., Zhang K., Qin Y., Yu J. (2017). A simple and green ultrasonic-assisted liquid–liquid microextraction technique based on deep eutectic solvents for the HPLC analysis of sesamol in sesame oils. Analytical Methods.

[bb0290] Liu W., Zhang K., Yang G., Yu J. (2019). A highly efficient microextraction technique based on deep eutectic solvent formed by choline chloride and p-cresol for simultaneous determination of lignans in sesame oils. Food Chemistry.

[bb0295] Ma C.H., Liu T.T., Yang L., Zu Y.G., Wang S.Y., Zhang R.R. (2011). Study on ionic liquid-based ultrasonic-assisted extraction of biphenyl cyclooctene lignans from the fruit of Schisandra chinensis Baill. Analytica Chimica Acta.

[bb0300] Ma Y., Wang H., Wang R., Meng F., Dong Z., Wang G., Chen M. (2019). Cytotoxic lignans from the stems of Herpetospermum pedunculosum. Phytochemistry.

[bb0305] MacFarlane D.R., Pringle J.M., Johansson K.M., Forsyth S.A., Forsyth M. (2006). Lewis base ionic liquids. Chemical Communications.

[bb0310] Malczak I., Gajda A. (2023). Interactions of naturally occurring compounds with antimicrobials. Journal of Pharmaceutical Analysis.

[bb0315] Mangindaan B., Matsushita Y., Aoki D., Yagami S., Kawamura F., Fukushima K. (2017). Analysis of distribution of wood extractives in Gmelina arborea by gas chromatography and time-of-flight secondary ion mass spectrometry. Holzforschung.

[bb0320] Mattioli S., Dal Bosco A., Castellini C., Falcinelli B., Sileoni V., Marconi O., Benincasa P. (2019). Effect of heat- and freeze-drying treatments on phytochemical content and fatty acid profile of alfalfa and flax sprouts. Journal of the Science of Food and Agriculture.

[bb0325] Mattioli S., Ruggeri S., Sebastiani B., Brecchia G., Dal Bosco A., Cartoni Mancinelli A., Castellini C. (2017). Performance and egg quality of laying hens fed flaxseed: Highlights on n-3 fatty acids, cholesterol, lignans and isoflavones. Animal.

[bb0330] Michailidis D., Angelis A., Aligiannis N., Mitakou S., Skaltsounis L. (2019). Recovery of Sesamin, Sesamolin, and minor Lignans from sesame oil using solid support-free liquid-liquid extraction and chromatography techniques and evaluation of their enzymatic inhibition properties. Frontiers in Pharmacology.

[bb0335] Moss, G. P. (2000). Nomenclature of Lignans and Neolignans (IUPAC recommendations 2000). 72(8), 1493-1523. Doi:Doi:10.1351/pac200072081493.

[bb0340] Mustafa A., Turner C. (2011). Pressurized liquid extraction as a green approach in food and herbal plants extraction: A review. Analytica Chimica Acta.

[bb0345] Nadar S.S., Rao P., Rathod V.K. (2018). Enzyme assisted extraction of biomolecules as an approach to novel extraction technology: A review. Food Research International.

[bb0350] Nadeem, M., Taj Khan, I., Khan, F., Shah, M. A., & Niaz, K. (2020). Lignans and flavonolignans. In (pp. 98-116).

[bb0355] Nasiri M., Ahmadzadeh H., Amiri A. (2020). Sample preparation and extraction methods for pesticides in aquatic environments: A review. TrAC Trends in Analytical Chemistry.

[bb0360] Nikule H.A., Nitnaware K.M., Chambhare M.R., Kadam N.S., Borde M.Y., Nikam T.D. (2020). In-vitro propagation, callus culture and bioactive lignan production in Phyllanthus tenellus Roxb: A new source of phyllanthin, hypophyllanthin and phyltetralin. Scientific Reports.

[bb0365] Olmo-Garcia L., Bajoub A., Benlamaalam S., Hurtado-Fernandez E., Bagur-Gonzalez M.G., Chigr M., Carrasco-Pancorbo A. (2018). Establishing the phenolic composition of Olea europaea L. leaves from cultivars grown in Morocco as a crucial step towards their subsequent exploitation. Molecules.

[bb0370] Patyra A., Kołtun-Jasion M., Jakubiak O., Kiss A.K. (2022). Extraction techniques and analytical methods for isolation and characterization of Lignans. Plants.

[bb0375] Pedersen-Bjergaard S., Rasmussen K.E. (1999). Liquid−liquid−liquid microextraction for sample preparation of biological fluids prior to capillary Electrophoresis. Analytical Chemistry.

[bb0380] Pereira R.G., Nakamura R.N., Rodrigues M.V.N., Osorio-Tobón J.F., Garcia V.L., Martinez J. (2017). Supercritical fluid extraction of phyllanthin and niranthin from Phyllanthus amarus Schum. & Thonn. The Journal of Supercritical Fluids.

[bb0385] Piao J., Liu L., Wang S., Shang H.B., He M., Quan N., Li D. (2018). Magnetic separation coupled with high-performance liquid chromatography-mass spectrometry for rapid separation and determination of lignans in Schisandra chinensis. Journal of Separation Science.

[bb0390] Plaza M., Turner C. (2015). Pressurized hot water extraction of bioactives. TrAC Trends in Analytical Chemistry.

[bb0395] Polshettiwar V., Cha D., Zhang X., Basset J.M. (2010). High-surface-area silica nanospheres (KCC-1) with a fibrous morphology. Angewandte Chemie (International Ed. in English).

[bb0400] Qian, Z., Chen, J., Lei, Q., Tan, G., Zou, Y., Peng, G., Xie, J. (2022). doi:10.21203/rs.3.rs-2381002/v1.

[bb0405] Qin Y., Wang R.Q., Xing R.R., Yang L., Chen X., Hu S. (2023). Dispersive liquid-liquid microextraction based on a supramolecular solvent followed by high-performance liquid chromatographic analysis of lignans in Forsythiae Fructus. Journal of Separation Science.

[bb0410] Qin Z., Chang Y.-L., Chen Z.-M., Wang Y.-G., Fan W., Gu L.-B., Wang X.-D. (2024). A novel strategy for preparing lignan-rich sesame oil from cold-pressed sesame seed cake by combining enzyme-assisted treatment and subcritical fluid extraction. Industrial Crops and Products.

[bb0415] Ramos Payán M., Bello López M.Á., Fernández-Torres R., Callejón Mochón M., Gómez Ariza J.L. (2010). Application of hollow fiber-based liquid-phase microextraction (HF-LPME) for the determination of acidic pharmaceuticals in wastewaters. Talanta.

[bb0420] Rezaee M., Assadi Y., Milani Hosseini M.-R., Aghaee E., Ahmadi F., Berijani S. (2006). Determination of organic compounds in water using dispersive liquid–liquid microextraction. Journal of Chromatography A.

[bb0425] Rodríguez-García C., Sánchez-Quesada C., Toledo E., Delgado-Rodríguez M., Gaforio J.J. (2019). Naturally Lignan-Rich foods: A dietary tool for health promotion?. Molecules.

[bb0430] Saito M. (2013). History of supercritical fluid chromatography: Instrumental development. Journal of Bioscience and Bioengineering.

[bb0435] Shen Q., Wang H., Li S., Feng J., Song G., Zhang Y., Wang H. (2020). Development of a mesoporous silica based solid-phase extraction and ultra-performance liquid chromatography-MS/MS method for quantifying lignans in Justicia procumbens. ELECTROPHORESIS.

[bb0440] Socas-Rodríguez, B., Herrera-Herrera, A. V., Hernández-Borges, J., & Rodríguez-Delgado, M. Á. (2017). Estrogenic compounds in yogurt. In yogurt in health and disease prevention (pp. 451-472).

[bb0445] Song H., Chang K., Zhang L., Zhu W., Li Y., Hu H., Li L. (2022). Matrix solid-phase dispersion coupled with HPLC-UV for simultaneous extraction, purification and determination of six Lignans in Schisandra chinensis fruits. Journal of Chromatographic Science.

[bb0450] Svec F. (2017). Monolithic columns: A historical overview. Electrophoresis.

[bb0455] Sykłowska-Baranek K., Łysik K., Jeziorek M., Wencel A., Gajcy M., Pietrosiuk A. (2018). Lignan accumulation in two-phase cultures of Taxus x media hairy roots. Plant Cell, Tissue and Organ Culture (PCTOC).

[bb0460] Talapatra S.K., Talapatra B., Talapatra S.K., Talapatra B. (2015). Chemistry of plant natural products: Stereochemistry, conformation, synthesis, biology, and medicine.

[bb0465] Teo C.C., Tan S.N., Yong J.W.H., Hew C.S., Ong E.S. (2010). Pressurized hot water extraction (PHWE). Journal of Chromatography A.

[bb0470] Trivedi D.N., Rachchh N.V. (2022). Graphene and its application in thermoplastic polymers as nano-filler- a review. Polymer.

[bb0475] Tu X., Chen W. (2018). A review on the recent Progress in matrix solid phase dispersion. Molecules.

[bb0480] Wang J., Jiang B., Shan Y., Wang X., Lv X., Mohamed J., Sun J. (2020). Metabolic mapping of Schisandra chinensis lignans and their metabolites in rats using a metabolomic approach based on HPLC with quadrupole time-of-flight MS/MS spectrometry. Journal of Separation Science.

[bb0485] Wang L.-X., Wang H.-L., Huang J., Chu T.-Z., Peng C., Zhang H., Tan Y.-Z. (2022). Review of lignans from 2019 to 2021: Newly reported compounds, diverse activities, structure-activity relationships and clinical applications. Phytochemistry.

[bb0490] Wang, R.-Q., Ge, X., Chen, X., Hu, S., Yang, L., Li, D., & Bai, X.-h. (2019). Ballpoint tip-protected oil-in-salt liquid-phase microextraction with high performance liquid chromatography for the determination of magnolol and honokiol from cortex Magnoliae officinalis. Instrumentation Science & Technology, 48(3), 254–268. doi:10.1080/10739149.2019.1705480.

[bb0495] Wang Y., Zhang G., Chi X., Chen S. (2018). Green and efficient extraction of podophyllotoxin from Sinopodophyllum hexandrum by optimized subcritical water extraction combined with macroporous resin enrichment. Industrial Crops and Products.

[bb0500] Wang Y., Zhu J., Du X., Li Y. (2023). Simultaneous extraction and determination of Lignans from Schisandra chinensis (Turcz.) Baill. Via diol-based matrix solid-phase dispersion with high-performance liquid chromatography. Molecules.

[bb0505] Wen C., Zhang J., Zhang H., Dzah C.S., Zandile M., Duan Y., Luo X. (2018). Advances in ultrasound assisted extraction of bioactive compounds from cash crops–a review. Ultrasonics Sonochemistry.

[bb0510] Wu J., Wu X., Wu R., Wang Z., Tan N. (2021). Research for improvement on the extract efficiency of lignans in traditional Chinese medicines by hybrid ionic liquids: As a case of Suhuang antitussive capsule. Ultrasonics Sonochemistry.

[bb0515] Wu L., Yu L., Ding X., Li P., Dai X., Chen X., Ding J. (2017). Magnetic solid-phase extraction based on graphene oxide for the determination of lignans in sesame oil. Food Chemistry.

[bb0520] Wu Y., Wang H., Wang Y., Brennan C.S., Anne Brennan M., Qiu C., Guo X. (2020). Comparison of lignans and phenolic acids in different varieties of germinated flaxseed (Linum usitatissimum L.). International Journal of Food Science & Technology.

[bb0525] Xia Y., Li J., Zhang Z., Luo S., Liu S., Ma C., Li W. (2020). Decoding biomass recalcitrance: Dispersion of ionic liquid in aqueous solution and efficient extraction of lignans with microwave magnetic field. PLoS One.

[bb0530] Xu H., Sun L., Du Y., Duan W., Li W., Luo S., Pan G. (2024). Magnetic molecularly imprinted polymer combined with solid-phase extraction for purification of Schisandra chinensis Lignans. Polymers.

[bb0535] Xue J., Wang R.Q., Chen X., Hu S., Bai X.H. (2019). Three-phase hollow-fiber liquid-phase microextraction based on deep eutectic solvent as acceptor phase for extraction and preconcentration of main active compounds in a traditional Chinese medicinal formula. Journal of Separation Science.

[bb0540] Xue J., Yang L., Chen X., Bai X.H., Hu S. (2021). Vortex-assisted dispersive liquid-phase microextraction for the analysis of main active compounds from Zi-Cao-Cheng-qi decoction based on a hydrophobic deep eutectic solvent. Journal of Separation Science.

[bb0545] Yang L., Said R., Abdel-Rehim M. (2017). Sorbent, device, matrix and application in microextraction by packed sorbent (MEPS): A review. Journal of Chromatography B.

[bb0550] Yang Q., Zhang Z., Sun X.-G., Hu Y.-S., Xing H., Dai S. (2018). Ionic liquids and derived materials for lithium and sodium batteries. Chemical Society Reviews.

[bb0555] Yashaswini P.S., Sadashivaiah B., Ramaprasad T.R., Singh S.A. (2017). In vivo modulation of LPS induced leukotrienes generation and oxidative stress by sesame lignans. The Journal of Nutritional Biochemistry.

[bb0560] Yashin A.Y., Yashunskii D.B., Vedenin A.N., Nifant’ev N.E., Nemzer B.V., Yashin Y.I. (2018). Chromatographic determination of Lignans (antioxidants) in food products. Journal of Analytical Chemistry.

[bb0565] Yıldırım S., Cocovi-Solberg D.J., Uslu B., Solich P., Horstkotte B. (2022). Lab-in-syringe automation of deep eutectic solvent-based direct immersion single drop microextraction coupled online to high-performance liquid chromatography for the determination of fluoroquinolones. Talanta.

[bb0570] Zhang C., Xing H., Yang L., Fei P., Liu H. (2022). Development trend and prospect of solid phase extraction technology. Chinese Journal of Chemical Engineering.

[bb0575] Zhang, F., Wang, X.-d., Li, K., Yin, W.-t., Liu, H.-m., Zhu, X.-l., & Hu, P. (2024). Characterisation of flavourous sesame oil obtained from microwaved sesame seed by subcritical propane extraction. Food Chemistry: X, 21, 101087. doi:doi:10.1016/j.fochx.2023.101087. PMC10805642 38268846

[bb0580] Zhang M., Chen K., Hu Z., Shen Q., Wang H. (2018). PRiME pass-through purification of lignans in Silybum marianum and UPLC-MS/MS analysis. Journal of Chromatography. B, Analytical Technologies in the Biomedical and Life Sciences.

[bb0585] Zhang Y., Cai P., Cheng G., Zhang Y. (2022). A brief review of phenolic compounds identified from plants: Their extraction, analysis, and biological activity. Natural Product Communications.

[bb0590] Zhang Z., Dai Y., Xiao Y., Liu Q. (2023). Protective effects of catalpol on cardio-cerebrovascular diseases: A comprehensive review. Journal of Pharmaceutical Analysis.

[bb0595] Zhao X., Zhang N., Liu M., Deng F., Wu M. (2017). Purification and preparation of silybin and isosilybin by solid phase extraction. Chromatography.

[bb0600] Zhou J., Hu Y., Chen P., Zhang H. (2020). Preparation of restricted access monolithic tip via unidirectional freezing and atom transfer radical polymerization for directly extracting magnolol and honokiol from rat plasma followed by liquid chromatography analysis. Journal of Chromatography. A.

[bb0605] Zhou Y., Fuentes-Hernandez C., Shim J., Meyer J., Giordano A.J., Li H., Kippelen B. (2012). A universal method to produce low–work function electrodes for organic electronics. Science.

[bb0610] Zhou Y., Men L., Sun Y., Wei M., Fan X. (2021). Pharmacodynamic effects and molecular mechanisms of lignans from Schisandra chinensis Turcz. (Baill.), a current review. European Journal of Pharmacology.

[bb0620] Zhuang Y., Yu F., Ma J., Chen J. (2015). Graphene as a template and structural scaffold for the synthesis of a 3D porous bio-adsorbent to remove antibiotics from water. RSC Advances.

