# Supplementary material for: Trends in extraction and purification methods of Lignans in plant-derived foods
Source: Food Chem X. 2025 Feb 3;26:102249. doi: 10.1016/j.fochx.2025.102249 (PMC11848485; doi:10.1016/j.fochx.2025.102249)
Supplement: Supplementary file 1 — Fig. S1. Four monomers of lignans. Fig. S2. Six secondary structures of lignans [file mmc1.docx]

**Supplementary Materials**

**Methodology for article selection:** This review conducted literature searches using databases: PubMed and Google Scholar. The search terms input were: "lignans", "pretreatment", "purification", "extraction", "determination", "overview", "progress", "recent", "survey", "update", "summary", and "review" in various combinations. The inclusion criteria involved an initial screening of abstracts and introductions to identify studies on lignan analysis in plant-derived foods, after which a full-text analysis was undertaken to evaluate the pretreatment methods applied. The search was conducted on: January 18, 2025.


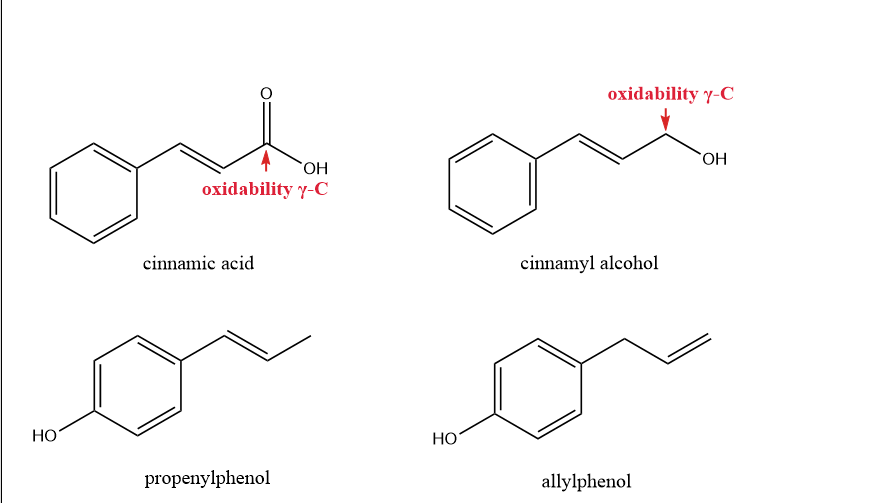


**Fig. S1.** Four monomers of lignans


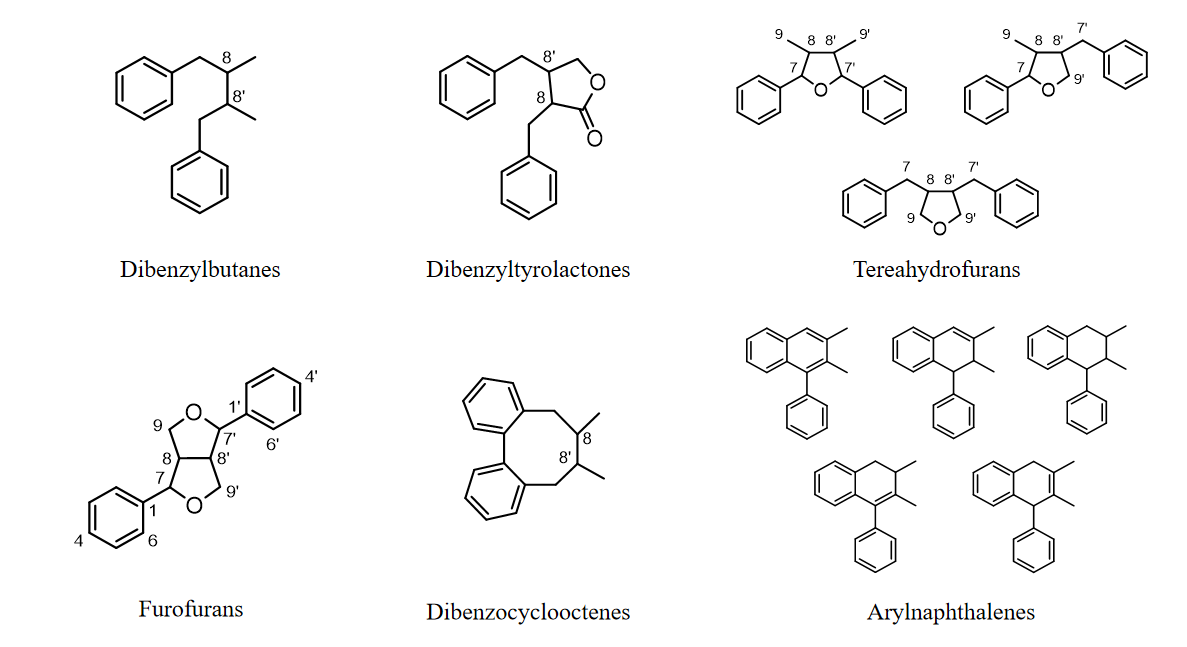


**Fig. S2.** Six secondary structures of lignans

**Table S1** Basic information of representative lignans

| Compound | CAS number | Boiling Point  (℃ at 760 mmHg) | Melting Point (℃) | Flash Point | Density (g/cm3) | Type of structural mother ring | Molecular Formula | Molecular weight  (g/mol) | Structure |
| --- | --- | --- | --- | --- | --- | --- | --- | --- | --- |
| Honokiol | 35354-74-6 | 400.1±40.0 | 87.5 | 184.0±21.9 | 1.1±0.1 | Neolignans | C_18_H_18_O_2_ | 266.334 |  |
| Magnolol | 528-43-8 | 401.0±40.0 | 99-101 | 184.5±21.9 | 1.1±0.1 | Neolignans | C_18_H_18_O_2_ | 266.334 |  |
| Niranthin | 50656-77-4 | 559.5±50.0 | N/A | 221.8±30.0 | 1.1±0.1 | Dibenzylbutanes | C_24_H_32_O_7_ | 432.507 |  |
| Phyllanthin | 10351-88-9 | 530.6±50.0 | 96 | 207.3±30.0 | 1.1±0.1 | Dibenzylbutanes | C_24_H_34_O_6_ | 418.523 |  |
| Pinoresinol | 487-36-5 | 556.5±50.0 | 121 | 290.4±30.1 | 1.3±0.1 | Furofurans | C_20_H_22_O_6_ | 358.385 |  |
| Podophyllotoxin | 518-28-5 | 597.9±50.0 | 183-184 | 210.2±23.6 | 1.4±0.1 | Arylnaphthalenes | C_22_H_22_O_8_ | 414.405 |  |
| Sesamin | 607-80-7 | 504.4±50.0 | 122-124 | 212.3±30.0 | 1.4±0.1 | Furofurans | C_20_H_18_O_6_ | 354.353 |  |
| Schisandrol A | 7432-28-2 | 576.7±50.0 | 128-129 | 302.6±30.1 | 1.1±0.1 | Dibenzocyclooctenes | C_24_H_32_O_7_ | 432.507 |  |

**Table S2** Comparison of the results of different pretreatment methods for lignans analysis

| Sample | Target analytes | Pretreatment | Total extraction yield | Recoveries | Time (min) | EF | Reference |
| --- | --- | --- | --- | --- | --- | --- | --- |
| *Forsythia suspensa* fruit | Arctiin, arctigenin, phillygenin, and phillyrin | DLLME based on SUPRAS | 8927.1-10426.8 μg/g | 96.5-104.8% | 0.5 | 6-170 | (Qin et al., 2023) |
| Health foods | Deoxyschizandrin, schisandrin A, schisantherin A | Solvent extraction based on IL | 2.97-5.59 μg/mg | 74.19-109.33% | 5 | n.d. | (Guan, Luo, Liang, & Yu, 2018) |
| Health foods | Eighteen multi-polar lignans | Solvent extraction based on HIL | 69 mg/g | 80.49-120.11% | 40 | n.d. | (Wu, Wu, Wu, Wang, & Tan, 2021) |
| Health foods | MG and HK | DI-SDME based on OIS | 10.32 mg/g | 84.5-99.8% | 30 | 73-76 | (Wang et al., 2019) |
| Health foods | MG and HK | DI-SDME based on BT | 10.32 mg/g | 84.5-99.8% | 30 | 73-76 | (Wang et al., 2019) |
| Health foods | MG and HK | DLLME | n.d. | 90.2-99.4% | 15 | 87-119 | (Guo, Pang, Zhang, Jiang, & Pang, 2013) |
| Health foods | MG and HK | DLLME based on SFOD | 131.7-141.5 μg/g | 88.9-105.4% | 40 | 229-252 | (Geng, Chen, Li, Bai, & Hu, 2020) |
| Health foods | MG and HK | D-μ-SPE | 29.21 μg/g | 99.6-99.7% | 2 | n.d. | (Chu et al., 2020) |
| Health foods | MG and HK | HFCF-UF | 1372-1606 μg/mL | 92.6-101.7% | 15 | n.d. | (An, He, Guo, & Dong, 2021) |
| Health foods | MG and HK | HF-LPME | 10.2-10.5 mg/g | 94.2-97.6% | 45 | 71 | (Xue, Wang, Chen, Hu, & Bai, 2019) |
| Health foods | MG and HK | HF-LPME based on DESs | 10.2-10.5 mg/g | 94.2-97.6% | 45 | 71 | (Xue et al., 2019) |
| Health foods | MG and HK | HF-LPME based on OIS | 1.14 μg/mL | 103.2-109.5% | 45 | 29.3-29.6 | (Li et al., 2018) |
| Health foods | MG and HK | MEPS | 2904.15 ng/mL | 93.0-101.73% | 10 | n.d. | (Zhou, Hu, Chen, & Zhang, 2020) |
| Health foods | MG and HK | SPE based on monolithic columns | 2904.15 ng/mL | 93.0-101.73% | 10 | n.d. | (Zhou et al., 2020) |
| Health foods | MG and HK | SPE based on RAMs | 2904.15 ng/mL | 93.0-101.73% | 10 | n.d. | (Zhou et al., 2020) |
| Health foods | MG and HK | VALLME | 10.2 mg/g | 83.5-108% | 4 | 197-210 | (Xue, Yang, Chen, Bai, & Hu, 2021) |
| Health foods | Phyllanthin and niranthin | SFE (adding ameliorants) | 12.36-37.21mg/g | n.d. | 33 | n.d. | (Pereira et al., 2017) |
| Health foods | Podophyllotoxin | SBE based on water | 0.4 mg/mL | 83.80% | 90 | n.d. | (Wang, Zhang, Chi, & Chen, 2018) |
| Health foods | Schisandrin A, schisandrin B, schisantherin A, schizandrol A, and schizandrol B | Solvent extraction based on DESs | 33.736 mg/g | 98.08-108.8% | 20 | n.d. | (Chen et al., 2024) |
| Health foods | Schisandrol A and angeloylgomisin H | D-μ-SPE based on PEI-MNPs | n.d. | 84.1-104.4% | 10 | n.d. | (Piao et al., 2018) |
| Health foods | Silybin and isosilybin | SPE | 933.33-970.59 mg/g | 94.4-101.4% | n.d. | n.d. | (Zhao, Zhang, Liu, Deng, & Wu, 2017) |
| *Justicia procumbens* L. | 6'-Hydroxy justicidin B, 6'-hydroxy justicidin A, justicidin B, chinensinaphthol methyl ether, justicidin C, and neojusticdin A | SPE based on KCC-1 | 0.63-0.81 mg/g | 83.4-96.1% | n.d. | n.d. | (Shen et al., 2020) |
| Olive oil by-product | Acetoxypinoresinol and pinoresinol | PLE | 49 mg/kg | n.d. | 20 | n.d. | (Cea Paze et al., 2019) |
| *S.chinensis* fruits | Deoxyschizandrin, schisandrin C, schisantherin A, schizandri, and γ-schizandrin | Solvent extraction based on ILs containing Lewis base | 4.12 mg/g | n.d. | 12 | n.d. | (Xia et al., 2020) |
| *S.chinensis* fruit | Schizandrol A, schisantherin A, schizandrin A, and schizandrin B | SPE based on MIPs | 0.29621 mg/mL | 93.13-98.75% | n.d. | n.d. | (Xu et al., 2024) |
| *S.chinensis* oil | Twenty-three lignans | SFE | 87.61 mg/g | 95.21% | n.d. | n.d. | (Gao, Wu, Cong, Xiao, & Ma, 2019) |
| *S.chinensis* oil | Twenty-three lignans | UAE | 67.73-80.94 mg/g | 93.24% | 30 | n.d. | (Gao et al., 2019) |
| Sesame oil | Sesamin, sesamol, and sesamolin | D-μ-SPE based on MGO | 8.19-11.25 mg/g | 85.48-93.78% | 4.5 | n.d. | (Wu et al., 2017) |
| Sesamol oil | Sesamin, sesamol, and sesamolin | DLLME based on phenolic DESs | 92.31-5099.73 mg/kg | 97.3-120% | 30 | n.d. | (Liu, Zhang, Yang, & Yu, 2019) |
| Sesame oil | Sesamin and sesamolin | LLE | 670.10-691.54 mg/mL | n.d. | 44 | n.d. | (Michailidis, Angelis, Aligiannis, Mitakou, & Skaltsounis, 2019) |
| Twenty-five green coffee | Lariciresinol, matairesinol, and secoisolariciresinol | EAE | n.d. | 87-97% | 120 | n.d. | (Angeloni et al., 2020) |
| Twenty-five green coffee | Lariciresinol, matairesinol, and secoisolariciresinol | Solvent extraction | n.d. | 83-93% | 30 | n.d. | (Angeloni et al., 2020) |
| *Zanthoxylum armatum* leaves | Asarinin, eudesmin, fargesin, kobusin, planispine-A, and sesamin | MAE | 5282-10217 μg/g | 94.8-109.3% | 15 | n.d. | (Bhatt, Sharma, Kumar, Sharma, & Singh, 2017) |
